# Systematic Review and Meta-Analyses of Mobilization Alarms for Prevention of Falls in Hospital

**DOI:** 10.1111/jgs.70651

**Published:** 2026-08-19

**Authors:** Terry P. Haines, Kelly Stephen, Ravi Manohar, Hanne Dolan, Meghan Campbell, Alison Bravington, Ronald I. Shorr, Dai Pu

**Affiliations:** 1 School of Primary and Allied Health Care & National Centre for Healthy Ageing, Monash University, Frankston, Australia; 2 Edson College of Nursing and Health Innovation, Arizona State University, Tempe, Arizona, USA; 3 Independent Researcher, London, UK; 4 NIHR Yorkshire & Humber Patient Safety Research Collaboration, Bradford Teaching Hospitals NHS Foundation Trust, Yorkshire, UK; 5 Geriatric Research Education and Clinical Center, Malcom Randall Department of Veterans Affairs Medical Center and Department of Epidemiology, University of Florida, Gainesville, Florida, USA

**Keywords:** accidental falls, alarms, geriatrics, hospital, low value care

## Abstract

**Background:**

Accidental falls are a common and costly adverse event among hospital patients. Mobilization alarms are used for their prevention however research findings vary between strong favorable effects and harmful effects. We sought to establish the effectiveness of mobilization alarms for the prevention of inpatient falls; identify effect modifiers and sources of design-related bias that may be confounding this field.

**Methods:**

Systematic review of English-language publications with no year limit restrictions using databases Medline, Embase, CINAHL, and Scopus; last searched on February 16, 2026. Two independent reviewers selected publications focused on alarm systems for the prevention of falls among adult hospital inpatients. Three reviewers extracted data related to study outcomes along with effect modifiers and potential sources of design-related bias. We followed PRISMA reporting guidelines. We undertook meta-analyses of RCTs and non-RCTs; examined potential effect modifiers and sources of design-related bias, then expanded the analyses to a broader range of factors in univariate and multiple meta-regression analyses, and exploratory analyses of time-series data to address the research objectives. The primary outcomes were rates of falls (ratio and mean difference), falls resulting in injury, and proportion of patients becoming fallers.

**Results:**

Pooled incidence rate ratio (95% CI) of falls when exposed to alarms when just considering RCTs (*n* = 13 comparisons, 11 studies) was 0.93 (0.83 to 1.04); and for injurious falls (*n* = 7, 6) was 1.00 (0.87 to 1.16); while mean difference (95% CI) in rate of falls/1000 OBD (*n* = 9, 8) was −0.20 (−1.03 to 0.64); and odds ratio for becoming a faller (*n* = 5, 5) was 1.11 (0.91 to 1.35). Multiple meta-regression analyses yielded results even more strongly indicating null effect, and consistently identified prospective registration as the most protective factor against studies reporting over-optimistic results.

**Conclusions:**

Routine, hospital-wide use of mobilization alarms appears ineffective for the prevention of inpatient falls.

## 1 | Introduction

Falls are one of the most common and costly adverse events in hospital settings [1]. Mobilization alarms are often used as a front-line approach to prevent falls in hospitals. A Cochrane review of randomized controlled trials (RCTs) in 2018 included three RCTs and concluded they were of uncertain effectiveness [2]. Another review in 2020 included four RCTs and concluded they were harmful, increasing falls by 20% [3]. However, use of mobilization alarms remains common [4], possibly driven by conflicting evidence favoring use of mobilization alarms presented in studies excluded from previous reviews.

Multiple studies published since 2022 [5–9] have cited favorable findings from older, non-RCT evaluations [10–12] as providing motivation for implementing mobilization alarm systems rather than discussing findings from the more recent reviews of RCTs [2, 3]. Decision-makers implementing alarms may be dismissing findings from previous RCTs based on differences in contextual factors such as type of alarm or patient population which may act as effect modifiers. They may also be preferring what is increasingly being referred to as “Real World Evidence” in the form of non-RCT, observational designs [13]. It is unclear whether reasonings such as these are misplaced in this field.

There is need for a clear picture of this evidence base to be formed. This research has three objectives: (i) generate pooled effect estimates for the effectiveness of mobilization alarms based on RCTs alone compared to RCTs and non-RCTs combined; (ii) identify/explore/quantify potential effect modifiers and sources of design-related bias in this field; (iii) generate pooled effect estimates that account for potential effect modifiers and sources of design-related bias. We considered using a conventional meta-analysis of RCTs alone would not address the information needs of decision makers in this context and simply be an update of previous reviews of RCTs which appear to have been ignored [5–9]. The desire to identify/explore/quantify potential effect modifiers and sources of design-related bias, and to generate pooled effect estimates that account for these aligns with the justifications for inclusion of non-RCTs alongside RCTs to provide an explicit evaluation of the weaknesses of available studies and to improve the translation of current evidence and design of future studies [14].

## 2 | Methods

### 2.1 | Design

The methods employed for this review were registered across two PROSPERO registrations (CRD42024493851—for RCTs; CRD42024508684—for non-RCTs). Several processes and frameworks for integrating non-RCTs and RCTs to guide healthcare decision-making have been described, which we have drawn from and expanded upon in developing our approach [14–17]. These include steps of (i) defining the research question and identifying systematically the evidence, (ii) extracting data, (iii) critical appraisal with classification of potential sources of effect modification and bias, (iv) quantifying and examining reasons for heterogeneity, and (v) interpretation of effect estimates. In completing this final step, we constructed a global probabilistic model through meta-regression that directly adjusted for each study’s parameter for its potential effect modification or design-related bias [15, 17].

### 2.2 | Data Sources

We conducted searches across Medline, Embase, CINAHL and Scopus databases. The last date of searching was February 2026. A justification for this approach and detailed example of the search strategy is provided (Supplementary Material S1).

### 2.3 | Study Selection

We included English language publications focused on adult hospital populations (> 18 years) without limitations on year of publication. We excluded studies focused on alarms/monitoring systems that did not have the purpose of falls prevention and studies conducted in hospital outpatient departments or pediatric wards. We included studies describing a range of alarm types based upon the stated purpose being to prevent falls, not just detect when falls occurred. Studies that evaluated alarms as a part of broader, multifactorial programs were included in this review.

### 2.4 | Data Extraction

Two reviewers [R.M., K.S.] independently screened titles and abstracts, and then conducted full-text review with discrepancies regarding study inclusion arbitrated by a third reviewer [T.P.H.]. Data extraction was undertaken by three reviewers with cross-checking between reviewers given the complexity and range of data presentation approaches [K.S., R.M., T.P.H.].

We sought to maximize the value of data available in literature identified. We reached out to authors to seek data that would have aided in our analyses.

### 2.5 | Risk of Bias Classification

The Cochrane Risk-of-bias tool for Randomized trials (RoB 2) and Cochrane Risk Of Bias In Non-randomized Studies of Interventions (ROBINS-I V2) were applied independently by two reviewers for each research report, drawing from a pool of reviewers [D.P., R.M., H.D., K.S., M.C.] so no author of an included paper was involved with its assessment.

### 2.6 | Outcomes

We extracted summative data for our clinical outcomes related to the rate of falls/1000 occupied bed days (OBDs), the rate of falls with an injury per 1000 OBD, and where available, incidence rate ratios and mean differences in these rates with 95% CIs. We also extracted summative data on the proportion of patients who became fallers or were non-fallers during their admission on the study wards and odds ratios with 95% CIs. Other data extracted from papers were driven by the search for potential effect modifiers and sources of design-related bias (Supplementary Material S2).

### 2.7 | Data Synthesis

The Cochrane Handbook chapter on including non-randomized studies on intervention effects highlights that it is not always possible to pre-specify all decisions about the methods employed. Included in this were calculations undertaken to generate estimates of effect from studies where these effect estimates could be calculated [14]. The set of analytic principles that governed this and study-specific decisions are presented (Supplementary Materials S3 and S4). We used the term “exploratory” to demarcate other analyses that were not pre-specified. Random effects meta-analyses were undertaken for each outcome to address the first research objective. Funnel plots were used to investigate potential for publication bias. Exploratory subgroup analyses excluding studies with a mean age < 60 were performed.

Univariate meta-regression analyses were used to identify potential effect modifiers and sources of design-related bias. Factors included were based upon those described in our Prospero registrations. These are listed, along with their justification, and how they were applied in data collection/analysis (Supplementary Material S2). Some modification of what was pre-specified was required (e.g., coding of ward type as acute vs. subacute was expanded to include other ward typologies). However, two entirely new factors were added, based on review of included papers. These were whether mobilization alarms were “implemented and analysed at the ward-level”, and whether there was industry involvement/sponsorship/relationship with the investigation (Supplementary Material S2). Some outcomes planned for inclusion (e.g., alarm response times) were not able to be included due to too infrequent reporting.

Analyses were performed to understand two issues in greater depth. The first was pre-planned and examined the nature of dose–response relationships between the amount of alarm use and reduction in falls outcomes. We used dose–response, mixed effects meta-analyses following previous recommendations to consider linear models, quadratic models, and restricted cubic spline models [18, 19].

The second was exploratory, and examined the relationship between individual control and intervention phase data points relative to other data points in that phase using studies where time-series data were presented (minimum three data points per phase). Under ordinary conditions, we would expect the final data points in an intervention phase and first data points of a control phase to be distributed with half above and half below the mean of their respective phases, assuming a step-change in fall outcomes with the intervention. However, if data collection periods were being manipulated to be inclusive of more favorable data points, we might expect to see more high fall rate observations at the beginning of control phases, and more low fall rate observations at the end of intervention phases. We graphically presented results of intervention and control phases from these studies as standardized units within the study control or intervention phase to examine this. This calculation for a control phase point was:

$$\frac{\text{Control phase measurement minus mean of measurements within that stydy’s control phase}}{\text{Standard deviation of measurements within that study’s control phase}}$$

Available time-series data were presented to examine whether the first and last data points, or mean of first three and last three of the control and intervention phases respectively, were consistently higher or lower than the rest of that phase, and whether the magnitude of these standardized values was different based on whether data had been drawn from prospectively registered studies or ones that were not prospectively registered where the ability to manipulate data collection periods is greater.

We finally constructed multiple meta-regression models, commencing with variables with a significant association identified in univariate meta-regression analyses with addition or removal of other variables until a model that minimized the Akaike Information Criterion (AIC) was identified [20]. Multilevel, mixed-effects, meta-regression analyses employing maximum likelihood were used so that AIC could be calculated and guide model selection. Variables that would lead to listwise deletion of observations were not included as AIC cannot be compared between models using different sets of data [21]. The final model had independent variables recoded so that the constant term from the model represented the expected effect of the intervention if evaluated using conditions that minimized bias.

Statistical analyses were undertaken using Stata SE version 18.0 (College Station, Texas) with meta-analytic techniques using Restricted Maximum Likelihood estimators for between-study variance in random effects models.

## 3 | Results

Our search strategy resulted in 1447 hits of which 129 were reviewed full text. Another three papers were identified through additional reference list checking or were prior known by the authors (Figure 1). Fifty-six publications reporting 60 comparisons were identified. Characteristics of included trials are presented (Table 1, Supplementary Material S5). Contrasting comparisons from RCTs to those that were not revealed non-RCTs were more likely to be conducted in the USA, to have been published since 2016, involve use of video-based alarms, while RCTs were more likely to be prospectively registered, have a parallel control group, use multiple sources for falls data, to investigate alarms with pressure sensitive devices, and to have non-zero control condition dose. Ratings of risk of bias are presented (Supplementary Material S6). A majority of RCTs were identified as high risk of bias, and non-RCTs at critical risk. Bias in measurement outcome (relying on incident reports alone) was a common source of risk elevation.

The pooled incidence rate ratio (95% CI) of falls when exposed to alarms when just considering RCTs (*n* = 13 comparisons) was 0.93 (0.83 to 1.04); and for injurious falls (*n* = 7) was 1.00 (0.87 to 1.16); while mean difference (95% CI) in rate of falls/1000 OBD (*n* = 9) was −0.20 (−1.03 to 0.64); and odds ratio for becoming a faller (*n* = 5) was 1.11 (0.91 to 1.35); with statistical heterogeneity very low in each case (*I*^2^ = 0.00%) (Figure 2A–D). However, results favored use of alarms when all studies were pooled but with evident statistical heterogeneity (*I*^2^: 25%–73%, Figure 2) indicating need to further investigate this heterogeneity. Exploratory subgroup analyses excluding studies with mean age < 60 years did not substantively change these findings (Supplementary Material S7).

Funnel plots contained asymmetry indicating publication bias across outcomes particularly for non-RCTs with 7/48 non-RCT contrasts beyond the *p* < 1% contour line favoring the intervention for the incident rate ratio of rate of falls outcome (Supplementary Material S8). Univariate meta-regression analyses identified several factors indicating design-related bias. Prospective registration was associated with less favorable results to the intervention in 3/4 outcomes (Table 2), as was having a parallel control group in 2/4 outcomes. Smaller study size (larger standard errors) was associated with more favorable results to the intervention in 2/4 outcomes. Older patient age was the only effect modifier identified for more than one outcome. Dosage data presentations between studies were heterogeneous, meaning that caution should be applied when interpreting these (Supplementary Material S9).

Exploratory analyses of time-series data used monthly, ward-level data from two prospectively registered studies (3 comparisons) that were both RCTs, and ward or hospital level data from 15 studies (16 comparisons) that were not prospectively registered, and non RCTs. Data in the latter case were available fortnightly, monthly, quarterly, four monthly or yearly. First and last standardized values examined for the prospectively registered studies all approximated the null value (Supplementary Materials S10 and S11). This pattern was similar for trials that were not prospectively registered for measures related to the control phase. However, for the intervention phase, effect estimates of the last and the mean of the last three data points of the rate of falls outcome were substantially lower, and the rate of injurious falls to an even greater extent. These findings indicate that either the intervention is becoming increasingly effective in the last or last three data collection periods (an effect not observed in data from the prospectively registered RCTs) or that intervention data collection periods were ceased in studies that are not prospectively registered after highly favorable datapoints had been observed.

Multiple meta-regression analyses (Table 3) again consistently identified prospective registration to provide less favorable results for the intervention (3 of 4 outcomes), while smaller study size (larger standard errors) provided results more favorable to the intervention (2 of 4 outcomes). No effect modifiers (including type of alarm or ward type) of those that were entered into multiple meta-regression analyses had associations that were significant. Constant terms from multiple meta-regression models indicated mobilization alarms are likely to have a null effect across all four outcomes, with an adjusted incidence rate ratio (95% CI) 1.02 (0.86 to 1.20) for rate of falls, an adjusted mean difference of 0.25 (−0.80 to 1.30) for rate of falls, an adjusted incidence rate ratio of 1.01 (0.87 to 1.16) for rate of falls with injury, and odds ratio of 1.09 (0.92 to 1.29) for proportion becoming a faller.

## 4 | Discussion

We conclude that mobilization alarms do not appear to have an effect on falls in hospital. The pooled point estimate of mean difference in fall rates from RCTs of 0.2/1000 OBDs indicates a 30-bed ward operating at full occupancy would only prevent approximately 2 additional falls per year with use of alarms. There appeared to be an absence of clinically meaningful effects sufficient to justify routine use of mobilization alarms [22] regardless of which falls outcome was examined or whether we considered effects calculated using conventional meta-analysis of RCTs or multiple meta-regression adjusting for effect modifiers and sources of design-related bias.

These results differ from previous reviews [2, 3]. This is potentially due to inclusion of a much greater number of RCTs facilitated by calculation of effect estimates from data presented in reports. It may also be due to inclusion of RCTs where the primary outcome was a type of fall (e.g., bedside falls) rather than total falls, and by inclusion of RCTs where mobilization alarms have been provided as part of multifactorial intervention programs.

Our introduction highlighted that decision-makers may set aside pooled effect estimates from RCTs due to effect modifiers limiting their generalizability to the context of the decision-maker. However, meta-regression analyses including up to 60 comparisons found few effect modifiers influenced reported results for most fall outcomes. Failure to identify significant effect modifiers or subpopulations may be a consequence of a lack of effect, but also a lack of statistical power. For example, the effect point estimate for the rate of falls/1000 OBD outcome was particularly favorable on dementia wards, but was not a significant association as only one non-RCT study focused on this specific setting. Thus, some caution is required when interpreting our findings regarding effect modifiers where low numbers of studies had or did not have the factor in question.

Mean subject age was the only effect modifier found to be associated with two or more of our outcomes. This would typically indicate that mobilization alarms may be more effective in older patient populations. However, this finding appears to have been driven by results from non-RCT studies that had low mean age where mobilization alarms appeared to increase the rates of falls (Supplementary Material S8). We also note that prospectively registered studies had a mean age of 79 years compared to 72 years in other studies, and that pooled effects from prospectively registered studies were null findings across all outcomes.

Our introduction also highlighted decision-makers may be preferring non-RCTs as “Real World Evidence”. Funnel plots indicating high likelihood of publication bias among non-RCTs, and evidence that the duration of intervention periods may be being manipulated/cut-off following collection of highly favorable data points in non-RCTs provide compelling evidence that this “Real World Evidence” should not be preferred by decision-makers. This concurs with previous simulation studies which indicate the ability to manipulate the duration of intervention and control phases increases the risk of a spurious significant finding from (normally) 5% to 27% [23]. However, using randomization alone was not found to be protective from over-optimistic findings in this review. The factor most likely to be protective was use of prospective trial registration. This finding concurs with previous simulation work drawing historical control data and concurrent controlled data from within a RCT dataset [24]. These simulations found that although there was an increase in amount of variability in outcomes, systematic bias was not evident. This indicates that it may not be the lack of randomization itself that introduces systematic biases of considerable magnitude, but other factors concurrent with a non-RCT design that is not prospectively registered.

There are three key implications of our findings. First, there appears to be an absence of clinically meaningful reductions in falls outcomes from use of mobilization alarms. Second, researchers describing evidence hierarchies or seeking to conduct meta-analyses in this and other fields should consider whether trials were prospectively registered as much, or potentially more, than just whether randomization was used. Third, hospital decision-makers should reconsider the utility of basing decisions on studies where a full description of the methods is not stated prior to collection of the data. It is understandable that systemic pressures encourage the use of readily available retrospective data rather than data collection at prospectively planned timepoints. But the consequence of this is that decision-making can be based on biased data and false assumptions, as the success of improvement initiatives is likely to naturally vary across time and context, and in relation to staff attitudes and behaviors [25]. A preconceived rationale for data collection is crucial to avoid bias, even when conducting quality improvement projects [26], and prospective registration for studies that are not RCTs was observed in this review [27].

This investigation did have limitations and left some issues un-resolved. Included RCTs were conducted on a mixture of acute and/or subacute wards. It is possible that use of mobilization alarms on specialty wards (e.g., dementia-specific) may yield different results. Calculation of effect estimates for some studies required use of assumptions such as 100% bed occupancy in order to calculate denominators for falls rates not presented in text. We also assumed that multifactorial intervention programs had no confounding effect on falls outcomes. These assumptions were considered reasonable; the latter in particular as there is only one intervention with proven effectiveness in this field (one-to-one, behavior change-based patient education) [28], and this intervention was not concurrently provided with mobilization alarms in any of the studies identified.

Future summative research investigating dose–response relationships in this field may be worthwhile, particularly if more prospectively registered trials that have investigated multiple dosage levels within the one study are published to enable network analysis [29]. Future research is also warranted to see whether our findings are replicated in other areas of hospital falls prevention, prevention of other adverse outcomes in hospitals, and even beyond the hospital setting.

## Supplementary Material

Supplement-1

Supplement-2

Additional supporting information can be found online in the Supporting Information section. Supplementary Material S1 Search strategy justification and examples of outputs from CINAHL and Embase. Supplementary Material S2: Potential effect modifiers and sources of design-related bias, their justification for inclusion, and application in data collection/analysis. Supplementary Material S3: Analytic principles when calculating effect estimates from included studies. Supplementary Material S4: Study-level analytic decisions. Supplementary Material S5: Study dataset. Supplementary Material S6: Risk of bias assessment. Supplementary Material S7: Subgroup analyses excluding studies with mean age < 60 years. Supplementary Material S8: Funnel plots across study outcomes by subgroup of whether trial is an RCT or non-RCT. Supplementary Material S9: Dose–response curve for all studies. Supplementary Material S10: Time-series data analyses. Supplementary Material S11: Comparison of standardised falls and falls with injury rate data from prospectively registered and non-prospectively registered studies at beginning of control/eliminated alarms condition or end of intervention/high alarms use conditions. Data S1: jgs70651-sup-0002-DataS1.xlsx.

## Figures and Tables

**FIGURE 1 |:**
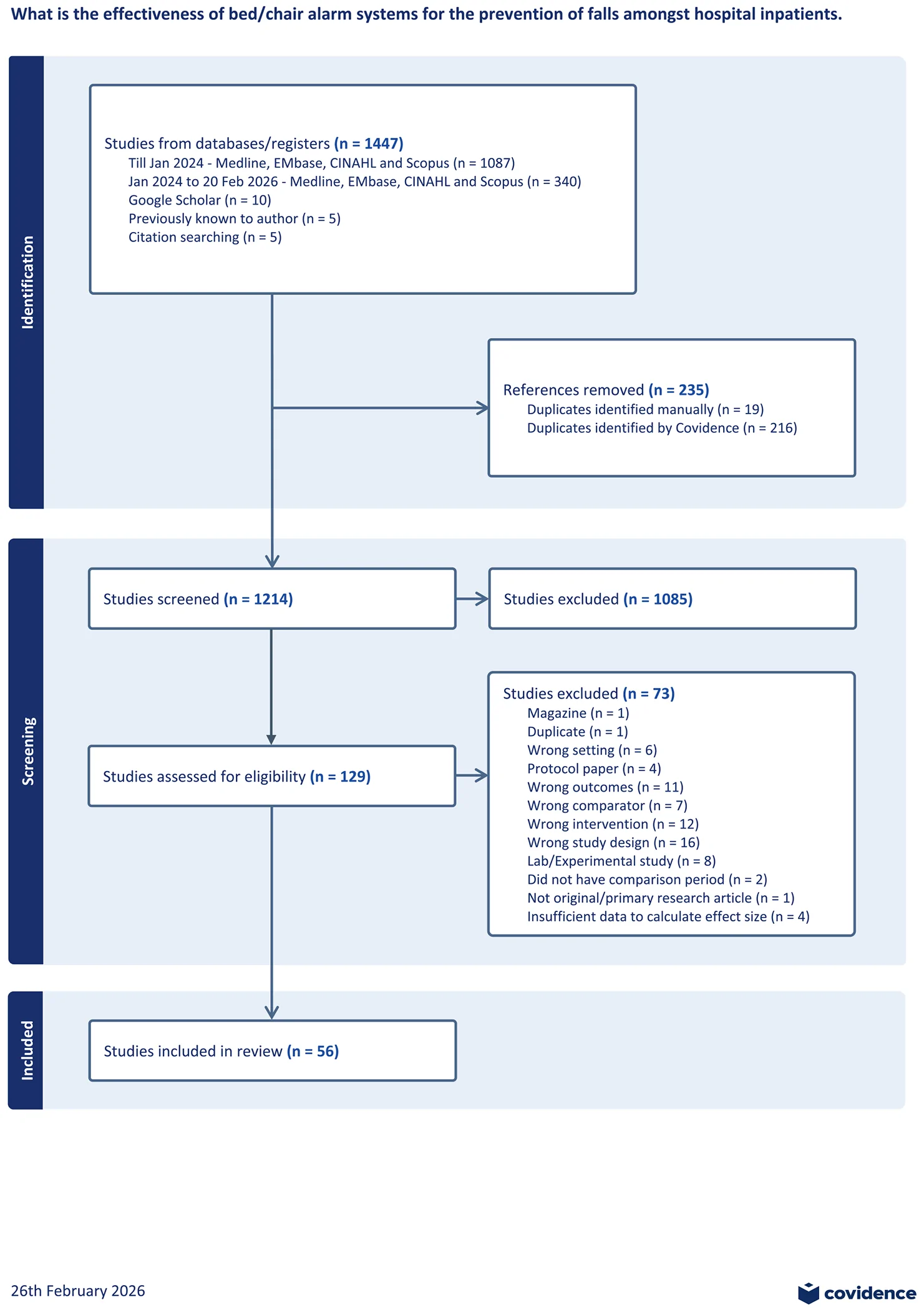
PRISMA flow chart.

**FIGURE 2 |:**
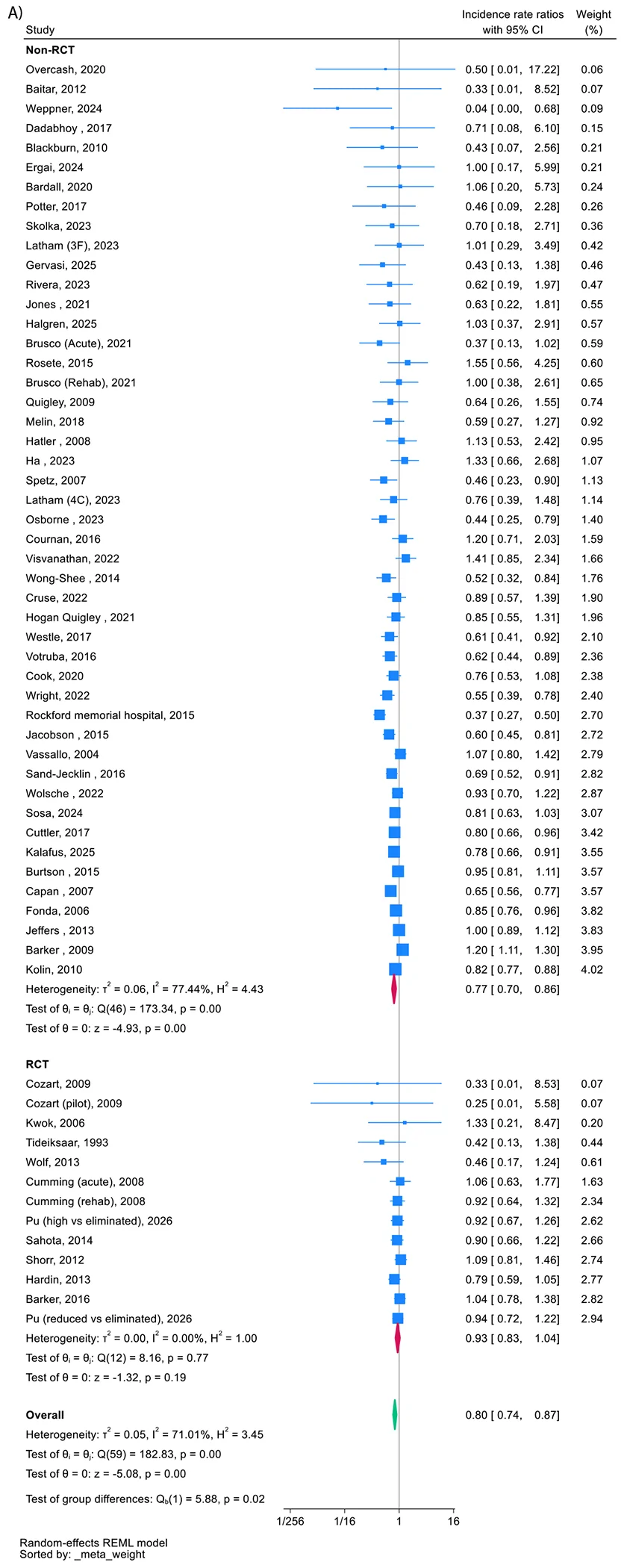

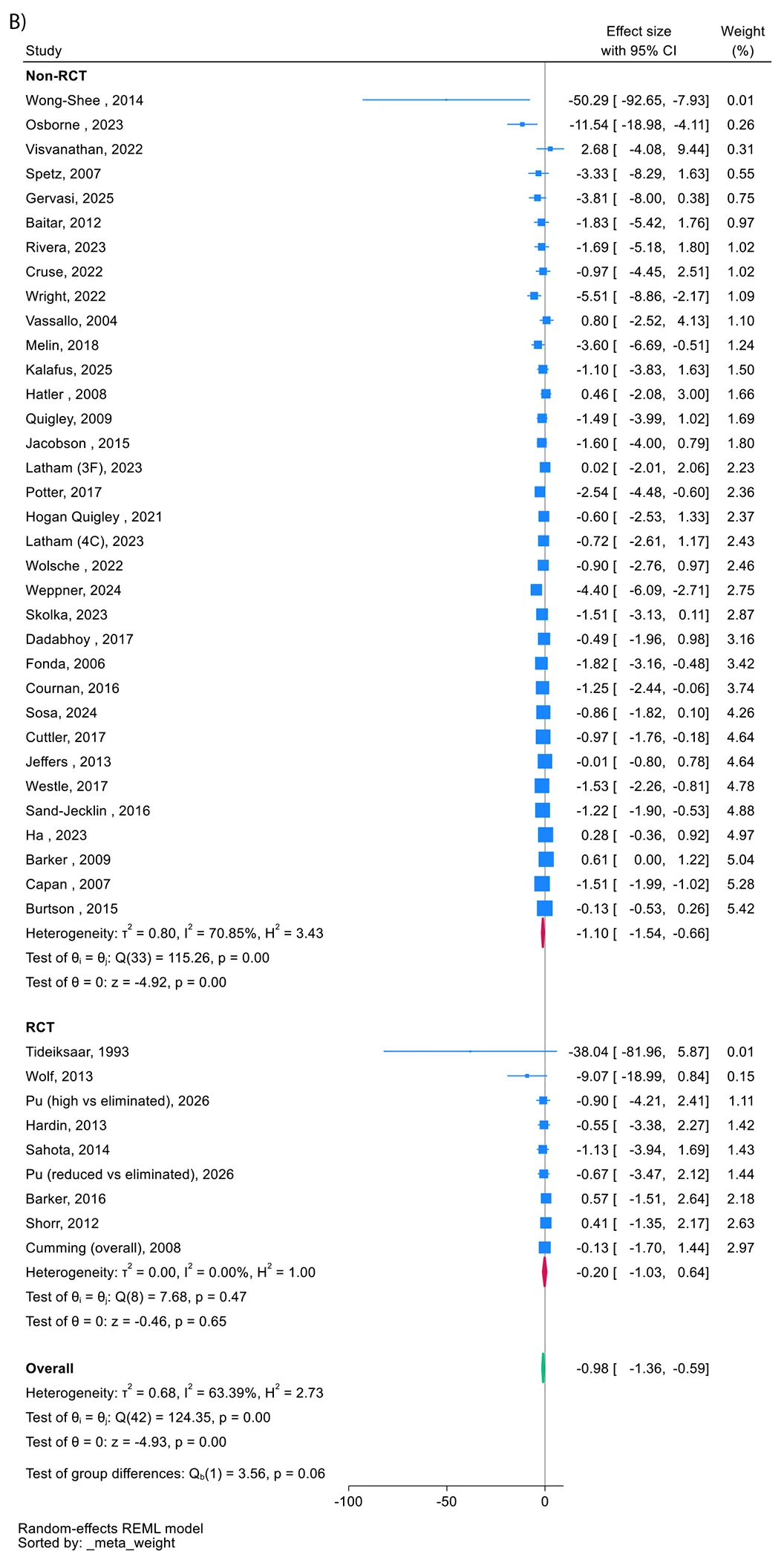

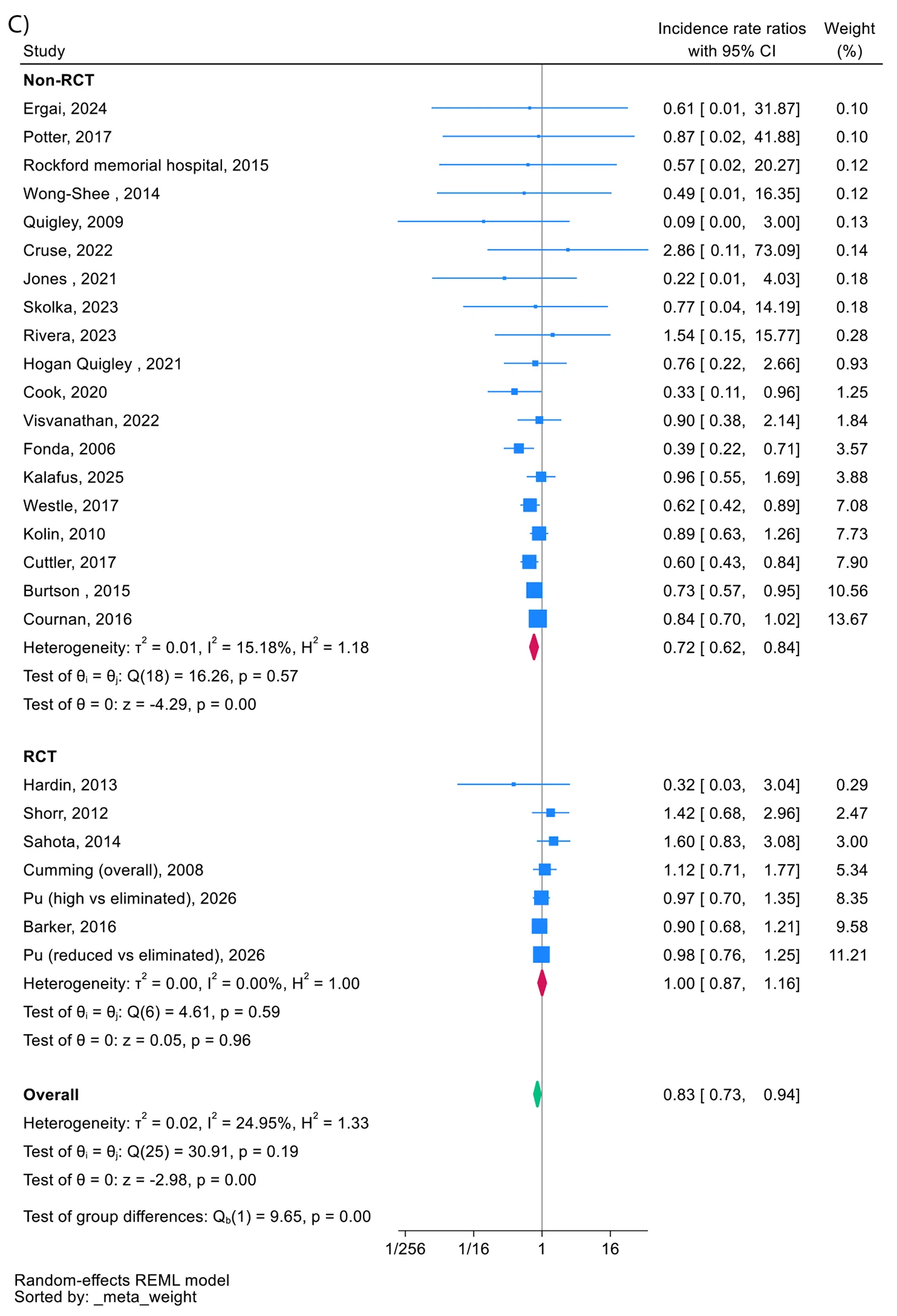

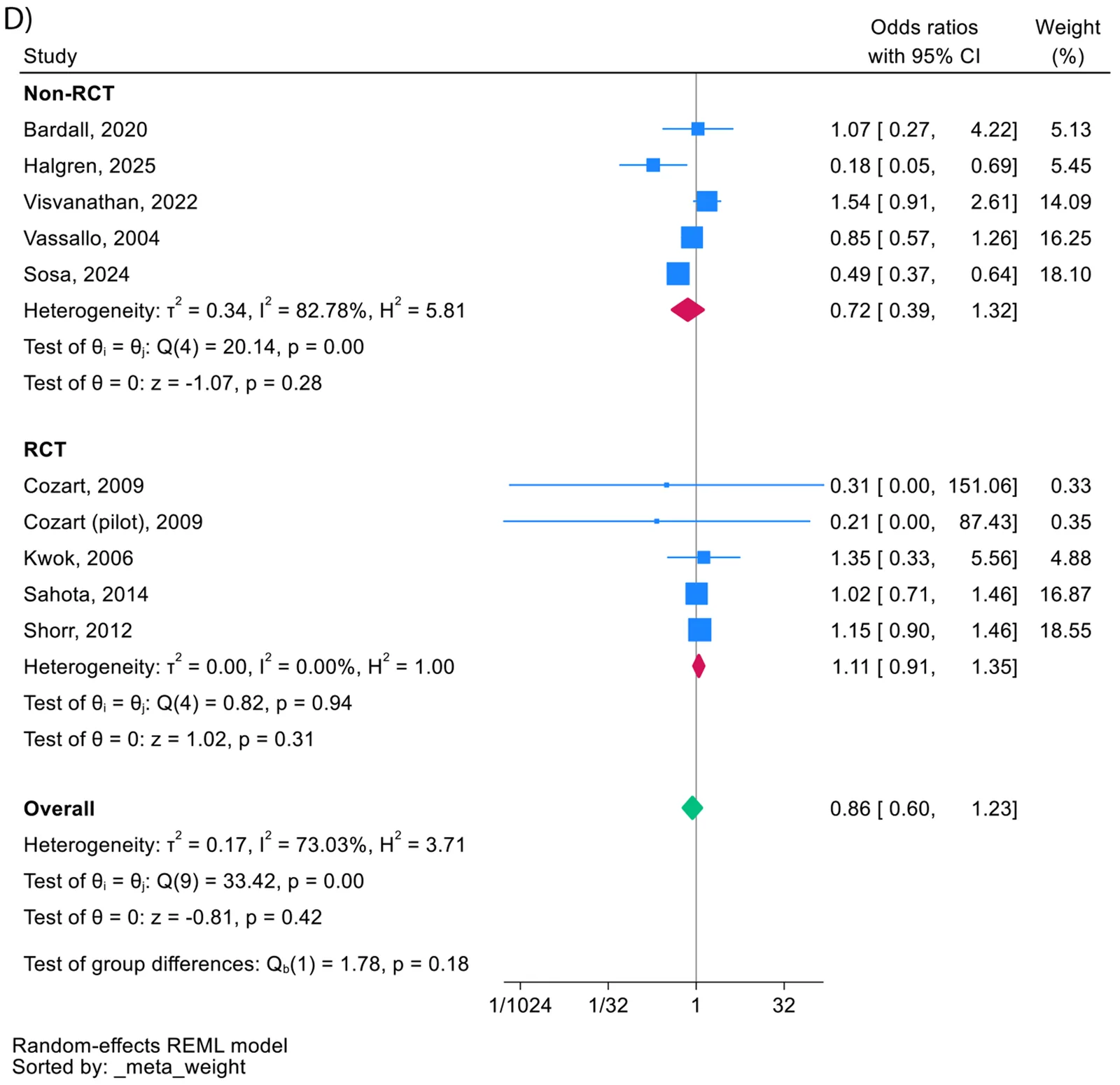
(A) Rate of falls (Incidence rate ratio). (B) Rate of falls (Mean difference). (C) Rate of falls with injury (Incidence rate ratio). (D) Proportion of fallers (Odds ratio).

**TABLE 1 |:** Characteristics of included comparisons.

Comparison characteristics	All comparisons	Comparisons from non-RCTs	Comparisons from RCTs
*n*: comparisons (studies)	61 (56)	48 (45)	13 (11)
Prospective registration—*n* (%)	8 (13%)	1 (2%)	7 (54%)
Parallel control group—*n* (%)	22 (36%)	9 (19%)	13 (100%)
Incident reports alone—*n* (%)	50 (82%)	44 (92%)	6 (46%)
Blinded data analyst—*n* (%)	2 (3%)	1 (2%)	1 (8%)
Blinded data collector—*n* (%)	1 (2%)	0 (0%)	1 (8%)
Industry relationship	7 (11%)	5 (10%)	2 (15%)
Publication type			
Peer review journal paper	48 (79%)	37 (77%)	11 (85%)
PhD thesis (no paper)	2 (3%)	0 (0%)	2 (15%)
Non-PhD thesis (no paper)	4 (7%)	4 (8%)	0 (0%)
Conference abstract (no paper)	7 (11%)	7 (15%)	0 (0%)
Year of publication			
≤ 2005	2 (3%)	1 (2%)	1 (8%)
2006–2015	24 (39%)	15 (31%)	9 (69%)
2016–2025	33 (54%)	32 (67%)	1 (8%)
> 2025	2 (3%)	0 (0%)	2 (15%)
Alarm type			
Pressure devices added to beds/chairs	16 (26%)	9 (19%)	7 (54%)
Devices imbedded in beds	3 (5%)	3 (6%)	0 (0%)
Video-based/vision technology	23 (38%)	22 (46%)	1 (8%)
Wearables	11 (18%)	8 (17%)	3 (23%)
Mixture of device typologies	6 (10%)	4 (8%)	2 (15%)
Unknown/not described	7 (11%)	7 (15%)	0 (0%)
Dose intervention—*n* (% of *n* with data)			
0%–3%	4 (10% of 40)	1 (4% of 27)	3 (23% of 13)
3.001%–6%	2 (5% of 40)	1 (4% of 27)	1 (8% of 13)
6.001%–15%	8 (20% of 40)	6 (22% of 27)	2 (15% of 13)
15.001%–99.999%	12 (30% of 40)	11 (41% of 27)	1 (8% of 13)
100%	14 (35% of 40)	8 (30% of 27)	6 (46% of 13)
Dose control—*n* (% of *n* with data)			
0%	34 (85% of 40)	26 (96% of 27)	8 (62% of 13)
0.001%–1%	3 (8% of 40)	0 (0% of 27)	3 (23% of 13)
> 1%	3 (8% of 40)	1 (4% of 27)	2 (15% of 13)
Alarms in isolation—*n* (%)	40 (66%)	32 (67%)	8 (62%)
Duration of control period—*n* (% of *n* with data)			
≤ 3 months	13 (25% of 52)	11 (24% of 46)	2 (33% of 6)
4–6 months	10 (19% of 52)	8 (17% of 46)	2 (33% of 6)
7–12 months	17 (33% of 52)	16 (35% of 46)	1 (17% of 6)
> 12 months	12 (23% of 52)	11 (24% of 46)	1 (17% of 6)
Duration of intervention period			
≤ 3 months	13 (25% of 52)	11 (24% of 46)	2 (33% of 6)
4–6 months	12 (23% of 52)	10 (22% of 46)	2 (33% of 6)
7–12 months	14 (27% of 52)	13 (28% of 46)	1 (17% of 6)
> 12 months	13 (25% of 52)	12 (26% of 46)	1 (17% of 6)
Control period duration minus intervention —*n* (% of *n* with data)			
Intervention ≥ 12 months longer	4 (8% of 52)	4 (9% of 46)	0 (0% of 6)
Intervention < 12 months longer	7 (13% of 52)	7 (15% of 46)	0 (0% of 6)
No difference	35 (67% of 52)	29 (63% of 46)	6 (100% of 6)
Control < 12 months longer	5 (10% of 52)	5 (11% of 46)	0 (0% of 6)
Control ≥ 12 months longer	1 (2% of 52)	1 (2% of 46)	0 (0% of 6)
Control to intervention period interruption — *n* (% of *n* with data)			
No interruption	41 (80% of 51)	35 (78% of 45)	6 (100% of 6)
≤ 3 months	3 (6% of 51)	3 (7% of 45)	0 (0% of 6)
4–6 months	2 (4% of 51)	2 (4% of 45)	0 (0% of 6)
7–12 months	3 (6% of 51)	3 (7% of 45)	0 (0% of 6)
> 12 months	2 (4% of 51)	2 (4% of 45)	0 (0% of 6)
Mean age—*n* (% of *n* with data)			
≤ 50 years	2 (9% of 23)	2 (13% of 16)	0 (0% of 7)
51–60 years	0 (0% of 23)	0 (0% of 16)	0 (0% of 7)
61–70 years	5 (22% of 23)	3 (19% of 16)	2 (29% of 7)
71–80 years	8 (35% of 23)	5 (31% of 16)	3 (43% of 7)
> 80 years	8 (35% of 23)	6 (38% of 16)	2 (29% of 7)
Proportion female—*n* (% of *n* with data)			
≤ 20%	2 (8% of 24)	1 (6% of 16)	1 (13% of 8)
21%–40%	2 (8% of 24)	2 (13% of 16)	0 (0% of 8)
41%–60%	16 (67% of 24)	12 (75% of 16)	4 (50% of 8)
61%–80%	3 (13% of 24)	1 (6% of 16)	2 (25% of 8)
81%–100%	1 (4% of 24)	0 (0% of 16)	1 (13% of 8)
Rate of falls in the control group—*n* (% of *n* with data)			
0–5 falls/1000 OBD	30 (61% of 49)	26 (65% of 40)	4 (40% of 10)
5.001–10 falls/1000 OBD	12 (24% of 49)	8 (20% of 40)	4 (40% of 10)
10.01–15 falls/1000 OBD	3 (6% of 49)	3 (8% of 40)	0 (0% of 10)
> 15 falls/1000 OBD	4 (8% of 49)	2 (5% of 40)	2 (20% of 10)
Ward type—*n* (%)			
Subacute care	10 (16%)	8 (17%)	2 (15%)
Acute care	37 (61%)	28 (58%)	9 (69%)
Mixed subacute and acute care	7 (11%)	5 (10%)	2 (15%)
Emergency department	1 (2%)	1 (2%)	0 (0%)
Palliative care/oncology	3 (5%)	3 (6%)	0 (0%)
Dementia care	1 (2%)	1 (2%)	0 (0%)
Intensive care	1 (2%)	1 (2%)	0 (0%)
Psychiatric care	1 (2%)	1 (2%)	0 (0%)
Percentage change sitter use			
Reduction ≥ 50%	4 (7%)	4 (8%)	0 (0%)
Reduction < 50%	4 (7%)	4 (8%)	0 (0%)
No change or no information present	50 (82%)	38 (79%)	12 (92%)
Increase < 50%	1 (2%)	1 (2%)	0 (0%)
Increase ≥ 50%	2 (3%)	1 (2%)	1 (8%)

**TABLE 2 |:** Univariate and multiple meta-regression analyses for potential effect modifiers and sources of design-related bias across each outcome.

Factor	Incidence rate ratio of falls (*n* = 60 comparisons, *n* = 56 studies)	Mean difference rate of falls/1000 OBD (*n* = 43 comparisons, *n* = 42 studies)	Incidence rate ratio of falls with injury (*n* = 26 comparisons, *n* = 25 studies)	Odds ratio of becoming a faller (*n* = 10 comparisons, *n* = 10 studies)
Study design a RCT	1.17 (0.96 to 1.43)	0.77 (−0.37 to 1.91)	1.37 (1.12 to 1.67)**	1.48 (0.72 to 3.07)
Ward-level implementation and analysis	1.16 (0.94 to 1.44)	0.40 (−0.77 to 1.58)	0.93 (0.66 to 1.30)	1.69 (0.90 to 3.17)
Prospective trial registration	1.32 (1.07 to 1.62)**	1.03 (−0.16 to 2.22)	1.38 (1.13 to 1.69)**	1.91 (1.21 to 3.02)**
Use of a parallel control group	1.14 (0.95 to 1.36)	0.71 (−0.13 to 1.56)	1.37 (1.12 to 1.66)**	2.25 (1.65 to 3.08)***
Use of incident reports alone	0.87 (0.71 to 1.06)	−0.36 (−1.47 to 0.76)	0.77 (0.63 to 0.93)**	0.56 (0.30 to 1.04)
Blinding of data analyst	1.34 (0.89 to 2.04)	0.55 (−2.43 to 3.52)	1.58 (0.90 to 2.79)	1.67 (0.79 to 3.52)
Blinding of data collector	1.12 (0.66 to 1.91)	−0.15 (−3.43 to 3.13)	1.96 (0.97 to 3.98)	1.23 (0.42 to 3.65)
Industry relationship	0.84 (0.63 to 1.12)	−1.01 (−3.12 to 1.10)	0.94 (0.40 to 2.21)	1.96 (0.73 to 5.23)
Publication mechanism				
Peer reviewed journal paper	1.31 (1.01 to 1.69)*	−0.35 (−1.62 to 0.92)	0.60 (0.05 to 6.72)	2.25 (0.73 to 6.93)
PhD thesis (no paper)	0.36 (0.04 to 3.43)	No comparisons	No comparisons	0.29 (< 0.01 to 23.17)
Non-PhD thesis (no paper)	1.08 (0.69 to 1.70)	0.54 (−1.15 to 2.23)	3.45 (0.13 to 89.57)	1.26 (0.24 to 6.60)
Conference abstract (no paper)	0.65 (0.49 to 0.88)**	0.09 (−1.72 to 1.90)	No comparisons	No comparisons
Year of publication	1.00 (0.98 to 1.01)	−0.04 (−0.11 to 0.02)	1.01 (0.99 to 1.03)	0.97 (0.93 to 1.02)
Alarm type				
Pressure devices added to beds/chairs	1.04 (0.86 to 1.27)	0.36 (−0.58 to 1.31)	1.08 (0.77 to 1.50)	1.53 (0.75 to 3.12)
Pressure-sensitive device imbedded in bed	1.08 (0.71 to 1.64)	0.43 (−1.21 to 2.08)	0.70 (0.48 to 1.03)	No comparisons
Video-based or infrared vision technology	0.91 (0.77 to 1.08)	−0.04 (−0.84 to 0.75)	0.99 (0.68 to 1.15)	0.43 (0.31 to 0.59)
Wearables	1.06 (0.83 to 1.35)	0.05 (−1.13 to 1.23)	1.29 (0.81 to 2.06)	1.47 (0.65 to 3.34)
Mixture of device typologies	1.09 (0.85 to 1.39)	0.44 (−0.93 to 1.81)	1.14 (0.89 to 1.47)	No comparisons
Unknown/not described	1.16 (0.85 to 1.57)	0.25 (−1.11 to 1.61)	1.07 (0.45 to 2.58)	1.47 (0.65 to 3.34)
Alarms in isolation	0.89 (0.75 to 1.06)	−0.46 (−1.26 to 0.33)	1.19 (0.90 to 1.58)	0.99 (0.38 to 2.56)
Smaller study size (larger standard error)	0.60 (0.37 to 0.98)*	−1.14 (−1.76 to −0.52)***	0.78 (0.43 to 1.45)	0.65 (0.20 to 2.14)
Duration of control (months)	(*n* = 51 comparisons) 1.01 (1.00 to 1.02)	(*n* = 39 comparisons) 0.04 (−0.01 to 0.09)	(*n* = 21 comparisons) 0.99 (0.97 to 1.00)	(*n* = 6 comparisons) 0.96 (0.84 to 1.09)
Duration of intervention (months)	(*n* = 51 comparisons) 1.01 (1.00 to 1.01)	(*n* = 39 comparisons) 0.03 (0.00 to 0.05)*	(*n* = 23 comparisons) 0.98 (0.97 to 1.00)*	(*n* = 6 comparisons) 0.96 (0.84 to 1.09)
Difference in duration (dichotomous)	(*n* = 51 comparisons) 1.03 (0.86 to 1.24)	(*n* = 39 comparisons) −0.40 (−1.23 to 0.43)	(*n* = 23 comparisons) 0.81 (0.64 to 1.01)	No comparisons
Control to intervention period interruption	0.91 (0.76 to 1.09)	−0.41 (−1.28 to 0.46)	0.96 (0.73 to 1.27)	1.14 (0.50 to 2.59)
Mean subject age	(*n* = 22 comparisons) 0.99 (0.98 to 1.00)*	(*n* = 17 comparisons) −0.05 (−0.08 to −0.02)***	(*n* = 7 comparisons) 1.00 (0.92 to 1.08)	(*n* = 7 comparisons) 1.02 (0.98 to 1.06)
Proportion female subjects	(*n* = 23 comparisons) 2.69 (0.98 to 7.38)	(*n* = 18 comparisons) 3.83 (−3.06 to 10.72)	(*n* = 9 comparisons) 0.12 (< 0.01 to 280.45)	(*n* = 8 comparisons) 21.55 (0.04 to 11518.73)
Rate of falls in the control group	(*n* = 50 comparisons) 1.00 (0.99 to 1.00)	(*n* = 43 comparisons) −0.17 (−0.29 to −0.05)**	(*n* = 22 comparisons) 1.01 (0.98 to 1.03)	(*n* = 8 comparisons) 1.02 (0.90 to 1.17)
Ward type/patient population				
Subacute wards	1.09 (0.85 to 1.39)	−1.12 (−2.42 to 0.17)	1.06 (0.73 to 1.54)	0.71 (0.30 to 1.69)
Acute wards	0.88 (0.74 to 1.05)	0.33 (−0.53 to 1.18)	0.90 (0.71 to 1.13)	0.90 (0.41 to 1.98)
Mixed subacute and acute wards	1.19 (0.95 to 1.48)	−0.16 (−1.07 to 1.38)	1.13 (0.89 to 1.43)	1.95 (0.73 to 5.23)
Emergency departments	0.94 (0.54 to 1.65)	No comparisons	0.39 (0.13 to 1.19)	No comparisons
Palliative care or oncology units	1.13 (0.58 to 2.21)	0.54 (−1.01 to 2.08)	No comparisons	No comparisons
Dementia care	0.67 (0.39 to 1.16)	−4.60 (−8.29 to −0.91)*	No comparisons	No comparisons
Intensive care	1.42 (0.59 to 3.38)	1.46 (−1.58 to 4.50)	No comparisons	No comparisons
Psychiatric care	1.33 (0.23 to 7.52)	No comparisons	No comparisons	1.26 (0.24 to 6.60)
Percentage change sitter use	1.02 (0.78 to 1.34)	0.15 (−1.43 to 1.74)	1.17 (0.88 to 1.55)	No comparisons

* *p* < 0.05.

** *p* < 0.01.

*** *p* < 0.001.

**TABLE 3 |:** Multiple meta-regression models minimizing Akaike Information Criterion using explanatory factors without missing data.

	Incidence rate ratio of falls (*n* = 60 comparisons, *n* = 56 studies)	Mean difference rate of falls/1000 OBD (*n* = 43 comparisons, *n* = 42 studies)	Incidence rate ratio of falls with injury (*n* = 26 comparisons, *n* = 25 studies)	Odds ratio of becoming a faller (*n* = 10 comparisons, *n* = 10 studies)
Prospective trial registration	1.24 (1.04 to 1.49)*	1.52 (0.38 to 2.66)**	1.53 (1.17 to 1.99)**	
Publication mechanism: Conference abstract	0.68 (0.52 to 0.89)**			
Ward type: Dementia	0.68 (0.44 to 1.06)	−3.26 (−6.91 to 0.39)		
Alarm type: Video-based			1.19 (0.91 to 1.54)	
Smaller study size (standard error mean difference falls/1000 OBD)	0.63 (0.40 to 0.99)*			
Smaller study size (larger standard error)		−1.23 (−1.84 to −0.61)***		
Ward type: Emergency department			0.50 (0.17 to 1.48)	
Parallel control group				2.25 (1.65 to 3.08)***
Constant term from recoded model^a^	1.02 (0.86 to 1.20)	0.25 (−0.80 to 1.30)	1.01 (0.87 to 1.16)	1.09 (0.92 to 1.29)
Residual *I*^2^%—Higgins-Thompson	49.24%	51.86%	0.00%	0.00%

* *p* < 0.05.

** *p* < 0.01.

*** *p* < 0.001.

a Recoded model constant represents: (i) Incidence rate ratio of falls rate when the study was prospectively registered, published in a location other than conference abstract, on a non-dementia specific hospital ward, and had a large sample size (standard error of incidence rate ratio = 0.1499 taken from Shorr et al.). (ii) Mean difference in rate of falls/1000 OBD when the study was prospectively registered, a RCT, had a large sample size (standard error of mean difference = 0.9 - taken from Shorr et al.) and on a non-dementia specific hospital ward. (iii) Incidence rate ratio of falls with injury when the study was prospectively registered, used an alarm type other than video-based, and took place in a ward other than emergency department. (iv) Odds ratio of becoming a faller when a parallel control group was used.

## Summary

- Key points
  - Routine, hospital-wide use of mobilization alarms was not associated with prevention of hospital inpatient falls in randomized trials, but was in non-randomized trials.
  - Publication bias and investigators ceasing intervention period data collection following a favorable data point appear to contribute to over-optimistic results in non-randomized studies.
  - There were few effect modifiers identified and prospective trial registration was the factor most consistently identified as providing protection against reporting of over-optimistic results when all studies were considered.
- Why does this paper matter?
  - Mobilization alarms are very commonly used across hospitals in the US and internationally. This paper has identified that routine, hospital-wide use of mobilization alarms does not produce clinically meaningful reductions in falls. This paper has also identified that researchers appear to be manipulating the duration of data collection periods as a means of generating more favorable results in studies that are not prospectively registered.
