# Supplementary material for: Systematic Review and Meta-Analyses of Mobilization Alarms for Prevention of Falls in Hospital
Source: J Am Geriatr Soc. Author manuscript; Available in PMC 2026 Oct 9. (PMC13651459; doi:10.1111/jgs.70651)
Supplement: Supplementary file 1 [file NIHMS2210303-supplement-Supplement-1.pdf]

Supplementary Material S1: Search strategy justification and examples of outputs from CINAHL and Embase

Justification:

Optimal search strategies for a given research question are context-specific.[1] In our search, we began with Medline and Embase, a combination that has previously been observed to identify 95% of relevant literature in one review methodology investigation[1] and 97% in another.[2] We added to this CINAHL, a discipline-specific database for nursing and allied health journals, and Scopus, a multidisciplinary journal. CINAHL is a rich source of nursing and allied health research which includes grey literature (thesis dissertations, conference proceedings, and book chapters) and has been reported to uniquely identify between 5% and 33% of relevant studies in nursing-specific qualitative research compared to MEDLINE, PubMed, Embase, and PsychINFO.[3] Additional grey literature were identified through searching Google Scholar, citation searching, and works previously known to authors. We also included reference list checking, Google Scholar searching, and used references known to authors to increase our yield.

References -
1) Goossen K, Hess S, Lunny C, Pieper D. Database combinations to retrieve systematic reviews in overviews of reviews: a methodological study. BMC medical research methodology. 2020 Jun 1;20(1):138.
2) Bramer WM, Rethlefsen ML, Kleijnen J, Franco OH. Optimal database combinations for literature searches in systematic reviews: a prospective exploratory study. Systematic reviews. 2017 Dec 6;6(1):245.
3) Wright K, Golder S, Lewis-Light K. What value is the CINAHL database when searching for systematic reviews of qualitative studies?. Systematic reviews. 2015 Jun 26;4(1):104.

Example of output: Search Strategy 1-

MEDLINE AND EMBASE

(bed* or chair* or mobil*) adj3 (alarm* or sensor* or system* or technolog*) OR

remote sensing technology/ or "electronic sensor*" OR

Beds/ or Clinical Alarms/ or electronic sensor/ or chair/ OR

remote sens* technol*

AND

(fall or falls or slip* or trip*) adj2 prevent* OR

(Accidental adj1 (fall or falls) or "in?patient falls" OR

Accidental Falls/

CINAHL

bed* or chair* or mobil* N3 alarm* or sensor* or system* or technolog* OR

"electronic sensor*" OR

remote sens* technol* OR

(MH "Equipment Alarm Systems") OR

(MH "Beds and Mattresses")

AND

(fall or falls or slip* or trip*) N2 prevent* OR

Accidental N1 (fall or falls) OR

inpatient falls OR

(MH "Accidental Falls") OR

Additional terms to capture alarms using video-monitoring

(video or visual or remote) N1 (monitor* or surveillanc* or observ*)

AND

(fall or falls or slip* or trip*) N2 prevent* OR

Accidental N1 (fall or falls) OR

inpatient falls OR

(MH "Accidental Falls") OR

SCOPUS

(bed* or chair* or mobil*) W/3 (alarm* or (sensor* or system* or technolog*) OR

"electronic sensor*" OR

remote sens* technol* OR

AND

(fall or falls or slip* or trip*) W/2 prevent* OR

Accidental W/1 (fall or falls) or in?patient falls

Search Strategy 2-Video monitoring on falls Prevention

MEDLINE AND EMBASE

(video or visual or remote) adj1 (monitor* or surveillanc* or observ*)

AND

"in?patient falls*" OR

(fall or falls or slip* or trip*) adj2 prevent* OR

(Accidental adj1 (fall or falls) OR

Accidental Falls/

Additional terms to capture alarms using video-monitoring

(video OR visual OR remote) W/1 (monitor* OR surveillanc* OR observ*)

AND

(fall or falls or slip* or trip*) W/2 prevent* OR

Accidental W/1 (fall or falls) OR

in?patient falls

Example of search output: CINAHL

Search History (12)

#	Searches	Results	Type	Runtime	Actions	Annotations
1	((bed* or chair* or mobil*) adj3 (alarm* or sensor* or system* or technolog*)).mp. [mp=title, abstract, heading word, drug trade name, original title, device manufacturer, drug manufacturer, device trade name, keyword heading word, floating subheading word, candidate term word]	27191	Advanced	0.00	Display Results More	
2	remote sensing technology/ or "electronic sensor*".mp. [mp=title, abstract, heading word, drug trade name, original title, device manufacturer, drug manufacturer, device trade name, keyword heading word, floating subheading word, candidate term word]	27036	Advanced	0.00	Display Results More	
3	Beds/ or Clinical Alarms/ or electronic sensor/ or chair/	12855	Advanced	0.00	Display Results More	
4	remote sens* technol*.mp. [mp=title, abstract, heading word, drug trade name, original title, device manufacturer, drug manufacturer, device trade name, keyword heading word, floating subheading word, candidate term word]	785	Advanced	0.00	Display Results More	
5	((fall or falls or slip* or trip*) adj2 prevent*).mp. [mp=title, abstract, heading word, drug trade name, original title, device manufacturer, drug manufacturer, device trade name, keyword heading word, floating subheading word, candidate term word]	15292	Advanced	0.00	Display Results More	
6	((Accidental adj1 (fall or falls)) or "in?patient falls").mp. [mp=title, abstract, heading word, drug trade name, original title, device manufacturer, drug manufacturer, device trade name, keyword heading word, floating subheading word, candidate term word]	4472	Advanced	0.00	Display Results More	
7	Accidental Falls/	58071	Advanced	0.00	Display Results More	
8	1 or 2 or 3 or 4	63829	Advanced	0.01	Display Results More	
9	5 or 6 or 7	67437	Advanced	0.03	Display Results More	
10	8 and 9	601	Advanced	0.01	Display Results More	
11	limit 10 to english language	580	Advanced	0.02	Display Results More	
12	limit 11 to dc=20240101-20260220	104	Advanced	7.82	Display Results More	

Example of search output: Embase Classic+Embase <Jan 2024 to 2026 February 16> Search update.

Search History (12)

#	Searches	Results	Type	Runtime	Actions	Annotations
1	((bed* or chair* or mobil*) adj3 (alarm* or sensor* or system* or technolog*)).mp. [mp=title, abstract, heading word, drug trade name, original title, device manufacturer, drug manufacturer, device trade name, keyword heading word, floating subheading word, candidate term word]	27191	Advanced	0.00	Display Results More	
2	remote sensing technology/ or "electronic sensor*".mp. [mp=title, abstract, heading word, drug trade name, original title, device manufacturer, drug manufacturer, device trade name, keyword heading word, floating subheading word, candidate term word]	27036	Advanced	0.00	Display Results More	
3	Beds/ or Clinical Alarms/ or electronic sensor/ or chair/	12855	Advanced	0.00	Display Results More	
4	remote sens* technol*.mp. [mp=title, abstract, heading word, drug trade name, original title, device manufacturer, drug manufacturer, device trade name, keyword heading word, floating subheading word, candidate term word]	785	Advanced	0.00	Display Results More	
5	((fall or falls or slip* or trip*) adj2 prevent*).mp. [mp=title, abstract, heading word, drug trade name, original title, device manufacturer, drug manufacturer, device trade name, keyword heading word, floating subheading word, candidate term word]	15292	Advanced	0.00	Display Results More	
6	((Accidental adj1 (fall or falls)) or "in?patient falls").mp. [mp=title, abstract, heading word, drug trade name, original title, device manufacturer, drug manufacturer, device trade name, keyword heading word, floating subheading word, candidate term word]	4472	Advanced	0.00	Display Results More	
7	Accidental Falls/	58071	Advanced	0.00	Display Results More	
8	1 or 2 or 3 or 4	63829	Advanced	0.01	Display Results More	
9	5 or 6 or 7	67437	Advanced	0.03	Display Results More	
10	8 and 9	601	Advanced	0.01	Display Results More	
11	limit 10 to english language	580	Advanced	0.02	Display Results More	
12	limit 11 to dc=20240101-20260220	104	Advanced	7.82	Display Results More	

Supplementary Material S2. Potential effect modifiers and sources of design-related bias, their justification for inclusion, and application in data collection/analysis.

Factor (type)	Justification	Applications in data collection/analysis
Dose of intervention (potential effect modifier)	Dose of intervention is widely recognised as a factor likely to influence the effect of interventions.[1]	Dose calculated based on data presented in report where available. A proportion was used to express dose (eg. proportion of patients who had alarms or proportion of time alarms were used) however the exact metric varied from study to study.
Study design RCT or not RCT (potential source of design-related bias)	Empirical research has demonstrated over 25 years ago that even well-designed quasi-experimental studies are more likely to report results that are favourable for an intervention and less likely to report harms than RCTs.[2]	Dichotomous classification based on whether patients or wards or hospitals were randomly allocated to receive the intervention or control condition.
Intervention implemented and analysed at the ward-level (potential effect modifier and source of design related bias)	Previous authors have noted the mechanism of mobilization alarms to prevent falls is to direct the attention of nursing staff toward an unsteady patient who is attempting to mobilize. However, the opportunity cost of providing attention to one patient when there is a limited amount of this resource comes at the expense of providing attention to another which can also lead to falls.[3] Thus, a RCT that allocates mobilization alarms to some subjects on a ward but not control group subjects who are treated on the same ward creates a control condition that receives a lower standard of care than the true counterfactual which existed prior to the trial commenced. Implementing alarms at the ward-level is not sufficient to address this concern, as some observational studies have done so but then selected patients from the same ward who did not receive the alarm to act as the control.[4,5] Previous authors have also noted that use of individual randomisation within a ward context creates situations where nursing staff seek to place alarms on patients who do not have alarms but are actually in the control group, which may introduce problems with intervention fidelity.[6]	Dichotomous classification based on whether alarms were implemented and analysed at the ward level or not.

Prospective trial registration (potential source of design-related bias)	Prospective trial registration was introduced by the International Committee of Medical Journal editors in 2005[7] to address the increasing concern that negative trials were not being published or that trial methods (eg. which outcome served as primary) were changed leading to a biased literature.	Dichotomous classification based on prospective trial registration with link to registration to allow verification needed to be provided in text.
Use of a parallel control group (potential source of design-related bias)	Previous meta-analyses of falls prevention interventions in hospitals identified favourable results were more frequently reported in studies with historical controls rather than concurrent controls[8]. Concurrent controls theoretically remediate temporal effects but may be more subject to sampling bias.	Dichotomous classification based on whether study reported concurrent or historical controls. Stepped wedge designs and multiple interrupted time series were treated as having a concurrent control.
Use of incident reports alone (potential source of design-related bias)	Multiple studies internationally have demonstrated underreporting of falls data if relying on a single data collection system, commonly at 25%-50%[9-12] but up to 90%.[13] This can be greatly improved by using multiple systems within the same study.[9,11,12] Where a single data collection system is used, there is potential for upregulation in reporting concurrent with introduction of a new falls prevention intervention leading to a systematic bias.	Dichotomous classification based on description of multiple sources of falls data collection being used. A single source was assumed if there was no mention of how falls data were collected.
Blinding of data analyst (potential source of design-related bias)	The data analyst has opportunity to make decisions not only about analytic techniques employed but also how much data is used which can inflate type I error rates.[14]	Dichotomous classification based on description of blinding of data analyst. No mention of blinding of data analyst led to assessment of none.
Blinding of data collector (potential source of design-related bias)	It is arguably impractical to blind hospital staff who record falls in this context, however, research personnel who perform the role of data collector, extracting data from medical records or other data systems and handle data after this point can potentially be blinded.	Dichotomous classification based on description of blinding of data collector. No mention of blinding of data collector led to assessment of none.
Industry relationship (potential effect modifier and source of design-related bias)	A potential "direct" realist factor but also "indirect" critical factor. Industry body involvement may lead to stronger tailoring of interventions to clinical context and systems, enhancing the "true effect" of the	Dichotomous classification based on description of relationships in conflict-of-interest declarations, project funding, acknowledgements, author affiliations, or in-text descriptions.

	intervention. However, industry involvement in research has also been shown to introduce bias directly and indirectly in some fields but not others.[15-17] Industry involvement may involve funding of research, provision of equipment as an in-kind contribution to the research, or at a discounted rate. Investigators may have some financial relationship with the mobilization alarm company. Investigators who contribute to design of the mobilization alarms may also have a financial stake in their intellectual property.	
Publication mechanism (potential source of design-related bias)	Inclusion of grey literature sources means that the level of scientific peer review scrutiny may differ across publications within the field. Scientific peer review may reduce bias in published results.	Dummy variables created based on whether the research was published in a peer review journal; not in a peer reviewed journal but in a PhD thesis; not in a peer reviewed journal but in a non-PhD research thesis; or not in a peer reviewed journal but in a conference abstract.
Year of publication (potential effect modifier and source of design-related bias)	A potential "indirect" indicator of both realist and critical factors. Earlier models of mobilization alarms may be less advanced compared to later models and may impact the effectiveness of mobilization alarm intervention. Research standards and strategies to enhance methodological rigor have also increased over time potentially reducing use of research approaches that enable bias.	Year of study publication used.
Alarm type (potential effect modifier)	Different approaches to triggering alarms may impact the effectiveness of mobilization alarm intervention.	Dummy variables created based on whether the alarms used pressure-sensitive devices added to beds / chairs; had devices imbedded in beds; used video-based or infrared vision technologies to trigger alerts; used wearable technologies (eg. accelerometers); a mixture of device typologies; or unknown / not described.
Alarms in isolation (potential effect modifier and source of design-related bias)	Use of alarms as a part of larger multifactorial intervention programs may make alarms appear more effective if other interventions are effective. It is also possible that other interventions (eg. staff education) may	Dichotomous classification based on description of intervention in text.

	enhance the "true effect" of mobilization alarms.	
Study size (potential source of design-related bias)	Asymptotically, larger samples are more likely to approximate the effect estimate in the population than smaller samples.	The standard error of the effect estimate was used. This was either taken directly from the research report or was calculated by the authors [TH] if a cluster-based design was used but not taken account of when effect estimates were described in the research report.
Duration of control period (potential effect modifier and source of design-related bias)	Asymptotically, larger samples are more likely to approximate the effect estimate in the population than smaller samples. Studies that have disinvested from use of alarms may require an adaptation period to adjust for this change in practice.	For ward-level intervention and analysis studies only. Months of control period measured as a continuous variable.
Duration of intervention period (potential effect modifier and source of design-related bias)	Asymptotically, larger samples are more likely to approximate the effect estimate in the population than smaller samples. Introduction of mobilization alarm interventions may be subject to a "wash-in" effect, where staff require an adaptation period to adjust for this change in practice. Conversely, a "waning effect" is possible, where initial enthusiasm for an intervention may wane over time.	For ward-level intervention and analysis studies only. Months of intervention period measured as a continuous variable.
Difference in duration of control and intervention period (potential source of design-related bias)	Difference in duration of intervention and control may indicate decisions by researchers to include some data points and not others.	For ward-level intervention and analysis studies only. Difference in months (rounded) of control and intervention period classified as a dichotomous variable (difference equals zero, or difference does not equal zero).
Interruption between control and intervention period (potential source of design-related bias)	A period of interruption in data collection to allow for the wash-in of an alarms intervention may remove delayed effects of the intervention from the dataset. Whether this is considered a source of bias or something that removes bias depends on the question being asked. Regardless it will likely impact the apparent effect estimate without actually changing whether the alarms are effective or not.	Presence of an interruption period classified as a dichotomous variable.
Mean age (potential effect modifier)	Increasing age and associated comorbidities that may influence the	Mean age of research subjects recorded. A mean was estimated where a median (IQR) was reported

	effectiveness of mobilization alarm interventions.	using the formula Mean = (lower quartile + 2 x median + upper quartile) / 4.[18]
Proportion female (potential effect modifier)	Male patients have been found to be at greater risk of falls while in hospital settings[19] and may have different motivations to take risks related to falls which may influence the effectiveness of mobilization alarm interventions.[20]	The proportion of research subjects identified as female recorded.
Rate of falls in the control group (potential effect modifier)	For the mean difference in rate of falls outcome, populations with higher rates of falls will have a higher mean difference with the same rate ratio reduction than populations with lower rates of falls.	Rate of falls per 1000 OBD entered as a continuous variable.
Ward type / patient population (potential effect modifier)	Hospital wards may differ in care philosophies and approaches based on their function (rehabilitation vs acute care), patient population, and stage of care in patient flow (eg. emergency department vs downstream wards).	Dummy variables created for research conducted on subacute wards; acute care wards; mixed subacute and acute care wards; emergency departments; palliative care or oncology units; dementia care; intensive care; and psychiatric care.
Sitter percentage change (potential effect modifier)	Some mobilization alarm studies specify the intent to reduce falls and reduce use of continuous patient observers (sitters). If sitters are effective in preventing falls (as indicated by synthesis of two non-RCTs),[21] then the percentage change in sitter use may impact falls.	Change in sitter use expressed as a percentage of control condition sitter use. i.e. (intervention sitter use minus control sitter use) divided by control sitter use. Reports that did not specify this information or provided insufficient information were classified as no change.

References:

1) Shim SR, Lee J. Dose-response meta-analysis: application and practice using the R software. Epidemiology and health. 2019 Mar 28;41:e2019006.
2) Weisburd D, Lum CM, Petrosino A. Does research design affect study outcomes in criminal justice? The Annals of the American Academy of Political and Social Science. 2001;578(1):50-70.
3) Spano-Szekely L, Winkler A, Waters C, Dealmeida S, Brandt K, Williamson M, Blum C, Gasper L, Wright F. Individualized fall prevention program in an acute care setting: an evidence-based practice improvement. Journal of nursing care quality. 2019 Apr 1;34(2):127-32
4) Hatler C. How intelligent are smart beds?. Nursing Management. 2008 Feb 1;39(2):20-2.
5) Spetz J, Jacobs J, Hatler C. Cost effectiveness of a medical vigilance system to reduce patient falls. Nursing economic$. 2007 Nov 1;25(6).
6) Timmons S, Vezyridis P, Sahota O. Trialling technologies to reduce hospital in-patient falls: an agential realist analysis. Sociology of health & illness. 2019;41(6):1104-19.
7) De Angelis C, Drazen JM, Frizelle FA, Haug C, Hoey J, Horton R, Kotzin S, Laine C, Marusic A, Overbeke AJ, Schroeder TV. Clinical trial registration: a statement from the International Committee of Medical Journal Editors. The Lancet. 2004 Sep 11;364(9438):911-2.

8) Hempel S, Newberry S, Wang Z, Booth M, Shanman R, Johnsen B, Shier V, Saliba D, Spector WD, Ganz DA. Hospital fall prevention: a systematic review of implementation, components, adherence, and effectiveness. Journal of the American Geriatrics Society. 2013 Apr;61(4):483-94.
9) Hill AM, Hoffmann T, Hill K, Oliver D, Beer C, McPhail S, Brauer S, Haines TP. Measuring falls events in acute hospitals—a comparison of three reporting methods to identify missing data in the hospital reporting system. Journal of the American Geriatrics Society. 2010 Jul;58(7):1347-52.
10) Sari AB, Sheldon TA, Cracknell A, Turnbull A. Sensitivity of routine system for reporting patient safety incidents in an NHS hospital: retrospective patient case note review. BMJ. 2007 Jan 11;334(7584):79.
11) Shorr RI, Mion LC, Chandler AM, Rosenblatt LC, Lynch D, Kessler LA. Improving the capture of fall events in hospitals: combining a service for evaluating inpatient falls with an incident report system. Journal of the American Geriatrics Society. 2008 Apr;56(4):701-4.
12) Sarkies MN, Bowles KA, Skinner EH, Haas R, Mitchell D, O'Brien L, May K, Ghaly M, Ho M, Haines TP. Do daily ward interviews improve measurement of hospital quality and safety indicators? A prospective observational study. Journal of Evaluation in Clinical Practice. 2016 Oct;22(5):792-8.
13) Cho I, Kwon JM, Choe W, Cho J, Park SH, Bates DW. Under-reporting of falls in hospitals: a multisite study in South Korea. BMJ Quality & Safety. 2025 Mar 26.
14) Haines TP, Beare R, Srikanth V. Research Note: Re-examining the issue of false positives (statistical type 1 errors) in the era of big data and high-frequency health measurement technologies. Journal of Physiotherapy. 2023 Oct 1;69(4):278-82.
15) Naci H, Dias S, Ades AE. Industry sponsorship bias in research findings: a network meta-analysis of LDL cholesterol reduction in randomised trials of statins. BMJ. 2014 Oct 3;349.
16) Fabbri A, Holland TJ, Bero LA. Food industry sponsorship of academic research: investigating commercial bias in the research agenda. Public Health Nutrition. 2018 Dec;21(18):3422-30.
17) King J, Yamey G, Smith R. Why journals should not publish articles funded by the tobacco industryForAgainst. BMJ. 2000 Oct 28;321(7268):1074-6.
18) Wan X, Wang W, Liu J, Tong T. Estimating the sample mean and standard deviation from the sample size, median, range and/or interquartile range. BMC Medical Research Methodology. 2014 Dec 19;14(1):135.
19) Haines TP, Hill AM. Inconsistent results in meta-analyses for the prevention of falls are found between study-level data and patient-level data. Journal of Clinical Epidemiology. 2011 Feb 1;64(2):154-62.
20) Haines TP, Lee DC, O'connell B, McDermott F, Hoffmann T. Why do hospitalized older adults take risks that may lead to falls? Health Expectations. 2015 Apr;18(2):233-49.
21) Greeley AM, Tanner EP, Mak S, Begashaw MM, Miake-Lye IM, Shekelle PG. Sitters as a patient safety strategy to reduce hospital falls: a systematic review. Annals of Internal Medicine. 2020 Mar 3;172(5):317-24.

Supplementary Material S3. Analytic principles when calculating effect estimates from included studies.

1. We sought to capture or calculate estimates of the incidence rate ratio of the rate of falls per 1000 OBD, the rate of injurious falls per 1000 OBD, the mean difference in the rate of falls per 1000 OBD, and the odds ratio in the proportion of patients who became fallers vs non-fallers during their admission.
2. We preferenced using data reporting "total falls" where available, and would use an alternate outcome (eg. falls by the bedside) if this was reported as the study's main/primary falls outcome and total falls were not available.
3. We preferenced using estimates of effect reported in metrics we were synthesising that were included in studies where available.
4. We calculated estimates of effect in metrics we were synthesising where they were not reported in individual papers using a variety of approaches depending on what data were available, preferencing approaches that most closely utilised the data presented in the paper and minimised assumptions.
   i. If multiple data points across comparison groups for numbers of falls or injurious falls were presented and number of occupied bed days; we used the Poisson regression command "poisson" in Stata to calculate the incidence rate ratio treating the occupied bed days data as an exposure variable, and the iri command to calculate the risk difference. Where hierarchical data were presented (data clustered in different wards/hospitals), we used clustering and robust variance estimates or mixed models to account for this.
   ii. If multiple data points across comparison groups for rates of falls or injurious falls were presented; we used the linear regression command "regress" in Stata to calculate risk differences. Where hierarchical data were presented (data clustered in different wards/hospitals), we used clustering or mixed models to account for this.
   iii. If a single data point across comparison groups for numbers of falls or injurious falls were presented and number of occupied bed days; we implemented the iri command in Stata[1] to calculate incidence rate ratios and risk differences[2].
   iv. If data related to the number of fallers and non-fallers were presented; we implemented the csi command in Stata[1] to calculate odds ratios[2].
5. Where data were incomplete, we would first perform calculations that would permit relevant data to be calculated (eg. calculating fall rates from number of falls and number of occupied bed days). If relevant data were still missing, we would make assumptions that would allow the analyses to proceed with what we considered reasonable validity (eg. we commonly assumed that a pre-post study on the same ward for the same duration pre and post would generate the same number of occupied bed days during this time if these data were not reported). Studies that reported wards or hospitals with the number of beds were assumed to have 100% occupancy unless otherwise stated). Assumptions regarding the distribution of falls per patient were also made based on language presented in text (eg. a study that reported "4 patients who had a fall", it was assumed each had a single fall).
6. Where data permitted break-down into more refined units (eg. results from acute wards and subacute wards presented separately, or for two wards with different dosage levels), we entered the data separately were possible to allow for more refined meta-regression and dose-response analyses respectively.

References:
1) StataCorp. 2023. *Stata Statistical Software: Release 18*. College Station, TX: StataCorp LLC.

2) Fleiss, J. L., B. Levin, and M. C. Paik. 2003. Statistical Methods for Rates and Proportions. 3rd ed. New York: Wiley.

Supplementary Material S4: Study-level analytic decisions.

RCTs	Data processing notes
Barker 2016[1]	Author did not respond to request for dose data. Dose data estimated from Figure 2 using PlotDigitizer[2].<br>Mean difference in falls rates was calculated using data from supplementary file.<br><br>Incidence rate ratio for falls rate and injurious falls rate calculated using poisson command in Stata to implement an exponential-mean model, robust variance estimate used, using the rates of falls or rate of injurious falls as the inputs. Data clustered by ward-pair.<br><br>Mean difference in rate of falls calculated using regress command in Stata[3] with data clustered by ward pair and robust standard errors used.
Cozart 2009[4]	Note that this thesis contains description of both a main trial and a prior pilot study. Both are included in this meta-analysis. Note also in both the pilot study (3% of sample) and main trial (~35% of sample) there were post randomization exclusions if people did not stay at least 7 days. In the pilot there were exclusions also.<br><br>For the pilot study, a two-tailed p-value and numbers of fallers in each group are presented. This information was used with the csi command in Stata to estimate the number of participants in each group overall.<br><br>Each person had only one fall based on the in-text description of each fall for the main trial. Length of stay not able to be estimated based on descriptions provided.<br><br>Incidence rate ratio for the rate of falls was calculated using the iri command in Stata, using the number of falls and number of participants as inputs making the assumption that participants in each group had the same OBDs. For the pilot randomized trial, an assumption was made that there were 14.5 participants in each group as there was block randomisation described and 1 participant was a post-randomization exclusion. However, the group of the exclusion was not specified.<br><br>Mean difference in rate of falls not able to be calculated.<br><br>Odds ratio for number of fallers calculated using csi command in Stata, using data reported in Table 9 as inputs.
Cumming 2008[5]	Extraction of data from this report complicated by the number of falls not being reported for each group, but the number of single, double and >double fallers. The paper reported that 49 patients / 2047 were recommended for alarms and 40 complied with their use. The intention-to-treat number (49) was used for the numerator when calculating dose.<br><br>Total number of occupied bed days estimated based on the multiplying the mean LOS per patient by the number of rehabilitation and acute patients per ward and by the number of wards reported in the paper (Table 2).<br><br>Number of falls in each group calculated using a simultaneous equation, based on total number of falls reported in text to be 381 (x+y=381), and the incidence rate ratio for falls between groups reported in paper as being 1.02, and the number of occupied bed days in the intervention group being 3631 and in the control being 3591 [(x/3631)/(y/3591)=1.02].

	Calculating the number of injurious falls was not able to be done this way as the total number of injurious falls was not revealed in the paper.<br><br>Incidence rate ratio for rate of falls presented in Table 4 used. Data separated by acute and rehabilitation wards to allow for this ward level-factor to be investigated in the critical-realist analyses.<br><br>Incidence rate ratio for rate of injurious falls presented in text for the overall study used (not able to be broken down by ward-type).<br><br>Mean difference in rate of falls for the overall study calculated using iri command in Stata, with the number of falls calculated through our simultaneous equations and the number of OBDs as inputs.<br><br>Standard deviation in rate of falls for the mean difference dose response analysis taken by pooling data presented separately for acute and rehabilitation wards presented in Table 2. The N for this is 2 given only 2 data points used.
Hardin 2013[6]	Author responded to email query about dose data however did not have the data available anymore. Data for dose taken from Table reporting frequency of alarm use amongst fallers in intervention and control wards under assumption this reflects broader population.<br><br>Extraction of data from this study complicated by use of cluster RCT design (5 clusters intervention and 5 clusters control). It appears a cluster-level analysis may have been conducted however, only a p-value was reported. It is not clear whether there was adjustment for pre-study falls rates, or differing OBDs between wards. A p-value of 0.106 was reported for a comparison of Poisson counts and a p-value of 0.1085 was able to be reproduced by our calculations indicating incidence rate ratios calculated are likely to be similar.<br><br>Incidence rate ratio for rate of falls and rate of falls with injury calculated using iri in Stata, using total number of falls, number of serious injuries, and OBDs presented in Table 6 as inputs.<br><br>Mean difference in rate of falls estimated using regress command in Stata, rate of falls at ward-level calculated from data in Table 2 as inputs.
Kwok 2006[7]	Table 4 provides data on number of participants in each group who “suffered a fall”. Assumption made that this indicates each is a single faller. Sample size provided, but not length of stay. “55.6% provided the intervention” used as dose.<br><br>Odds ratio calculated using csi command in Stata with OR option, using data in table 4.<br><br>Incidence rate ratio for rate of falls calculated using iri command in Stata, using number of falls reported in table 4, assuming each is a single faller and that overall OBDs were the same between groups.<br><br>Mean difference in rate of falls not able to be estimated.
Pu 2026[8]	Summative data from original dataset used for dose.<br>Effect estimates calculated using eliminated condition as reference valued using original dataset. Note that the main paper does not have these data given its disinvestment

	paradigm. This step has been taken specifically for this meta-analysis to align the research paradigm with other studies.<br><br>Incidence rate ratio for rate of falls and rate of injurious falls calculated using mepoisson command in Stata, using monthly number of falls or injuries as the outcome, intervention category and calendar month as fixed effects, OBDs as the exposure variable, random intercepts for ward-pair and ward (nested within pair) and robust variance estimates.<br><br>Mean difference in rate of falls was calculated using the margins post-estimation command following mepoisson. An approach based on a normal distribution was not used due to the skew in monthly falls data in this study.
Sahota 2014[9]	Bedside falls reported in this study. We have used this as overall falls not presented.<br>Adjusted incidence rate ratio for rate of falls and rate of injurious falls presented in Table 1 used.<br>Mean difference in rate of falls estimated using iri command in Stata, with the number of falls and the number of OBDs as inputs.
Shorr 2012[10]	Dosage values provided by author upon request.<br>Number of patients from appendix table 1.<br><br>Incidence rate ratio (95%CI) for rate of falls and rate of injurious falls presented in paper used.<br>Odds ratio (95%CI) for faller vs non-faller presented in paper used.<br>Mean difference in falls rate (95%CI) presented in paper used.
Tideiksaar 1993[11]	OBDs estimated from statement that experimental group utilised the system for 4425hours indicating 184.3 days.<br>OBDs in control assumed to be the same as intervention.<br><br>Incidence rate ratio for rate of falls calculated using iri in Stata, using number of falls presented in text, OBDs for intervention group presented in text, and estimated OBDs for control as inputs.<br><br>Mean difference in rate of falls estimated using iri command in Stata, using number of falls presented in text, OBDs for intervention group presented in text, and estimated OBDs for control as inputs.
Wolf 2013[12]	Data extraction from this study complicated by presentation of falls data in multiple ways. We have used the total number of falls.<br>We recreated a patient-level dataset based upon the description of the falls profile in text. We assumed equivalent length of stay per patient between groups, and used the number of OBDs for the study overall reported in text to guide these estimates. We made assumptions about the distribution of number of falls per patient so these figures aligned. We made assumption that the night time fallers were not the same as the day time fallers, though this is not clear from the paper.<br><br>Incidence rate ratio for rate of falls calculated using iri command in Stata, using number of falls reported in text and estimate of OBDs as input.<br><br>Mean difference in rate of falls estimated using iri command in Stata, with the number of falls and estimate of OBDs as inputs.
**Non-RCTs**	

Baitar 2012[13]	Length of stay median of 19 days reported in paper however range was 2 to 130 days. Given falls data was reported over the 6-month time-period, we estimate a 6-bed unit for 6 months (6 beds * 182 days) generates 1092 occupied days assuming 100% occupancy. This same assumption is made for the 6-month control period and that no alarms were used in that period.<br>Assumption that each faller had only one fall is made.<br><br>Incidence rate ratio for rate of falls calculated using iri command in Stata, using number of falls as described in text and estimated OBDs description as input.<br><br>Mean difference in rate of falls estimated using iri command in Stata, with the number of falls and estimated OBDs as inputs.
Bardall 2020[14]	Author presents data both overall and then again excluding seizure falls. Total falls data used for meta-analysis.<br>Number of falls presented but not OBD data. The number of patients in each group was presented (66 pre, 31 post) making assumption of equivalent amount of OBD in each group untenable. Table 1 appendix J presented number of fallers. Setting was psychiatric unit meaning no comparable study in this review to base LOS on. Decision to use 30-day length of stay based on psychiatric wards tending to have longer length of stay than acute[15].<br><br>Incidence rate ratio for rate of falls calculated using iri command in Stata, using number of falls based described in text and estimated OBDs description as input.<br><br>Odds ratio for fallers vs non-fallers calculated using csi command in Stata, using number of fallers and sample size described in text.
Barker 2009[16]	Data for the for the hospital-wide analysis (Table 3) used.<br>Fall injuries rather than falls with injury data presented in paper. Not used as each fall could have multiple injuries and is a different metric to what is being considered.<br><br>Incidence rate ratio for falls rate ratio calculated using poisson command in Stata, with the number of falls and number of OBDs per year (exposure variable) as inputs.<br>Mean difference in falls rate calculated using regress command in Stata, with the rate of falls each year as input.
Blackburn 2010[17]	Cannot calculate the number of OBDs or periods of time for exposure. Only data available is the number of falls pre (4/month) vs post (1.7/month).<br><br>Incidence rate ratio for falls rate ratio calculated using poisson command in Stata to implement an exponential-mean model, robust variance estimate used, using the rates of falls as the inputs. However, use of robust variance estimate with only two observations led to non-sensical standard error, so conventional standard error used.<br><br>Mean difference in fall rates unable to be calculated.
Brusco 2021[18]	Extraction of data from this study complicated by disinvestment paradigm and reporting of results for acute and rehabilitation wards separately. Dosage is different and reported separately for each. This allows us to treat this as two studies. We have recorded these on separate lines within the dataset and used the annotation Brusco (Acute) and Brusco (Rehab) when referring to these datasets. The reduced and eliminated conditions act as

	the control period in each respectively. Data for percentage change sitter use not able to be calculated for Rehab ward due to denominator being a zero (value zero change used).<br><br>OBD not able to be estimated. Duration of time reported but not ward size.<br><br>Incidence rate ratio for falls rate calculated using iri command in Stata using the number of falls, and weeks of observation as the inputs. Assumption that bed occupancy was equivalent across periods used.<br><br>Mean difference in rate of falls not able to be calculated
Burston 2015[19]	This report has both within site / pre-post comparison data, and data for comparison to the CALNOC benchmark which would be equivalent to parallel control. However, no data on use of video monitoring in CALNOC sites is provided, so does not provide a useful comparison for our purpose. No statistical analysis is provided for either, but sufficient data is provided for us to conduct analyses with.<br><br>Falls rate per 1000 OBD data taken from Figure 3. Falls with injury rate per 1000 OBD taken from Figure 4. Total number of occupied bed days estimated by multiplying size of facility (595 beds) by period of time.<br>Number of falls estimated by using rate of falls data and estimates of OBD data.<br>Incidence rate ratio for falls rate ratio calculated using poisson command in Stata to implement an exponential-mean model, robust variance estimate used, using the rates of falls as the inputs.<br>Mean difference in rate of falls calculated using regress command in Stata, using the rates of falls as the input.
Capan 2007[20]	No statistical analysis of data presented. The intervention narrative describes an evolution over time. 5 calendar years of quarterly falls rate data presented. Decision made to compare 2002 and 2003 to 2005 and 2006 as the intervention was rolled out in 2004, so it is not clear which quarters in 2004 were intervention and which were control.<br><br>OBDs calculated by multiplying facility size (357 beds) by number of days. Falls rate data from Figure 3 used. Number of falls estimated using falls rate and OBD estimate.<br><br>Incidence rate ratio for falls rate ratio calculated using poisson command in Stata to implement an exponential-mean model, robust variance estimate used, using the rates of falls as the inputs.<br><br>Mean difference in rate of falls calculated using regress command in Stata, using the rates of falls as the input.
Cook 2020[21]	This study is set in an emergency department. The falls rate is expressed as falls per 1,000 visits. Information is provided that 62 falls per year ~ 0.75 falls per 1000 visits. The raw number of falls were estimated from the falls rate data presented over the 2-year pre and post intervention periods. The difference between intervention and control periods of 27 falls and 10 injuries was noted and used in rounding estimates.<br><br>Incidence rate ratio for falls rate ratio calculated using poisson command in Stata to implement an exponential-mean model, robust variance estimate used, using the rates of falls as the inputs.

	Mean difference in fall rates unable to be calculated as data not available per 1000 OBD. This was not a problem for the rate ratio as the units for time cancel out in a ratio.
Cournan 2016[22]	Data for both the entire facility (115 beds) and for the traumatic brain injury unit presented. Mobile video monitors were not limited to the traumatic brain injury unit, so the whole of hospital data were used.<br><br>The results presented in the paper indicate monthly falls rate data were used in the t-test reported by the authors. This was successfully reproduced using the ttesti command in Stata. This was used for the mean difference calculation and the standard error of the mean difference.<br>A calculation of the incidence rate ratio was attempted using rounded estimates for the total number of falls based on the reported number of falls per month multiplied by the number of months. The command iri in Stata was used.
Cruse 2022[23]	Rate of falls/1000 OBD and number of falls presented in text. OBDs estimated using these data. Falls with injury number presented.<br><br>Incidence rate ratio for falls rate and falls with injury rate calculated using iri command in Stata using the number of falls or number of injuries respectively and estimated number of OBDs as the inputs.<br><br>Mean difference in rate of falls estimated using iri command in Stata using the number of falls and estimated number of OBDs as the inputs.
Cuttler 2017[24]	Dose unable to be estimated. Bed exit alarms were used during control period but not stated how often. In-built bed alarms were introduced during intervention phase, but no measure of how much they were activated. Study compared fall rates between Jan 2009 and Sept 2010 to Jan 2015 to Dec 2015. We have used these data. Falls and injuries per month estimated using plotdigitizer for time-based analyses. The removal of Q4 2010 (lowest fall rate from the potential comparison period) through to the end of 2014 is notable and may complicate the time-based analyses so may benefit from a sensitivity analysis restricting to just those studies with uninterrupted analyses between the control and intervention periods.<br><br>Incidence rate ratio for falls rate ratio and injurious falls rate ratio calculated using poisson command in Stata to implement an exponential-mean model, robust variance estimate used, using the quarterly rates of falls and injurious falls as the inputs.<br><br>Mean difference in rate of falls estimated using regress command in Stata on quarterly falls rate data from figure 2.
Dadabhoy 2017[25]	Data extraction complicated by presentation of data for the ACE unit (with intervention) compared to all of the hospital (of which ACE is a part) and also to an adjacent "comparison" unit. Given the ACE unit was a part of the overall hospital data, we have chosen to use the comparison to the adjacent unit.<br>Dose unable to be calculated, though description that bed alarms were used "heavily" provided.<br>OBDs for ACE ward calculated by multiplying number of patients by mean LOS.<br>Assumption of equivalent OBD on adjacent ward made.<br>Raw number of falls calculated using falls rate and OBD data, and then rounded.

	Incidence rate ratio for falls rate ratio calculated using poisson command in Stata to implement an exponential-mean model, robust variance estimate used, using the rates of falls as the inputs. However, use of robust variance estimate with only two observations led to non-sensical standard error, so conventional standard error used.<br><br>Mean difference in fall rates calculated using iri command in stata using rounded estimate of number of falls and estimates of OBDs as inputs.
Ergai 2024[26]	Unable to calculate dose. Description of implementing the video sitter in 41 units across 4 hospitals. But no information regarding number of beds provided.<br><br>Incidence rate ratio for falls rate calculated using poisson command in Stata to implement an exponential-mean model, robust variance estimate used, using the monthly rates of falls and injurious falls from Figure as the inputs. Robust standard error created with only two data points was non-sensical. Conventional standard error used.<br><br>Mean difference in fall rates unable to be calculated as data not available per 1000 OBD.
Fonda 2006[27]	There is 1 year of quarterly falls data presented as a baseline, and 2 years of post-intervention data. The intervention was phased in over a 3-month period at the end of the baseline year, however, these data were included as a part of the baseline. These data as presented have been retained for our analyses as these are the analyses as presented by the author. We further cannot clearly disarticulate these data from the quarterly presentation of data in this paper as this period covers two quarters.<br>Dose data unable to be calculated.<br>Data from box 3 used for analyses.<br>Incidence rate ratio for falls rate ratio and injurious falls rate ratios calculated using iri command in Stata using the number of falls, number of injuries and number of OBDs as the inputs.<br><br>Mean difference in rate of falls estimated using iri command in Stata using the number of falls and number of OBDs as the inputs.
Gervasi 2025[28]	Extraction of data from this report complicated by performance of several multiple-regression analyses with different p-values. The unadjusted analysis reported was used. It is noted that the unadjusted analysis reported the most conservative treatment effect of all those reported.<br>It is noted that this was a parallel control study comparing fall rates of those allocated to video surveillance with those not allocated to video surveillance who were being treated in the same units. Allocation was based on STRATIFY falls risk score.<br><br>Incidence rate ratio for rate of falls reported in text used.<br><br>Mean difference in risk of falls calculated using iri command in Stata, using number of falls and mean days “length of observation” per observation from Table 2 as inputs.
Ha 2023[29]	This is a parallel control group study with a rate of falls per month provided for each ward. However, the two wards were not the same size (control=58 beds, intervention =46 beds). OBDs were estimated by multiplying number of beds in each ward by 365 days. The number of falls per year was estimated by multiplying the number of falls per month by 12 then rounding. The rate of falls per 1000 OBD was estimated by dividing the rounded number of falls by the OBD*1000.

	Incidence rate ratio for falls rate ratio calculated using iri command in Stata using the number of falls and number of OBDs as the inputs.<br><br>Mean difference in rate of falls estimated using iri command in Stata using the number of falls and number of OBDs as the inputs.
Halgren 2025[30]	Extraction of data from this report complicated by focus on "repeat falls" after an unwitnessed initial fall as an outcome, where there was reporting to enable calculation of an odds ratio (see below). However, total falls rate data also presented for the whole sample to enable a comparison of incidence rate ratio of falls rate outcome. As the intervention was only provided to those who experienced an unwitnessed fall, the overall falls rate data may underestimate the treatment effect in this study, thus may benefit from sensitivity analyses. This also means two rows of data will be necessary in the dataset, one where the dose is unknown and ward-level intervention delivery and analysis is 1 (total falls outcome) and one where the dose is 1 and ward-level intervention delivery and analysis is zero1 (repeat faller outcome).<br><br>The statement "Rate of repeat falls following an unwitnessed initial fall decreased from 11 out of 34 (32.4%) preintervention, to 3 repeat falls out of 37 unwitnessed falls (8.1%) postintervention." Indicates the authors were talking about fall events here, but it is not completely clear that this also refers to the number of people who had the initial unwitnessed fall. No email addresses of authors have been able to be identified to confirm this. However, we have made the assumption that the term "rate of repeat falls" is referring to the number of fallers who became repeat fallers given the presentation of data as a fraction. This may benefit from sensitivity analyses.<br><br>Incidence rate ratio for falls rate calculated using poisson command in Stata to implement an exponential mean model, using the rate data presented in text.<br><br>Odds ratio of becoming a faller after an initial unwitnessed fall calculated using csi command in Stata using data in text.
Hatler 2008[31]	Study within ICU. The authors indicate the pre period is 6 months, but we are left to assume the post period is 6 months also as they do not say this directly. Falls data for all patients is presented along with those who received the intervention. Decision to use the overall falls data.<br>The authors indicate 10 of the 24 beds received devices, indicates dosage is 0.417. However, they also describe a consent process of which 102 patients provided consent, so it is not clear if the alarms were only turned on for that number of patients and that the actual dosage is lower. The 0.417 dosage figure will be used.<br>15 falls reported pre intervention with average LOS of 5.9 days compared to 17 falls post intervention with average LOS of 5.6 days.<br>OBDs estimated by 24 beds multiplied by 182 days.<br><br>Incidence rate ratio for falls rate ratio calculated using iri command in Stata using the number of falls and number of OBDs as the inputs.<br><br>Mean difference in rate of falls estimated using iri command in Stata using the number of falls and number of OBDs as the inputs.
Hogan Quigley 2021[32]	Dose estimated by 10 mobile video cameras across 244 beds. Occupied bed days estimated by number of beds multiplied by days assuming 100% occupancy.<br>Rates of falls per 1000 OBD reported, used to estimate raw number of events.

	Incidence rate ratio for falls rate and injurious falls rate calculated using poisson command in Stata to implement an exponential-mean model, robust variance estimate used, using the monthly rates of falls and injurious falls from Figure as the inputs.<br><br>Mean difference in rate of falls estimated using regress command in Stata on monthly falls rate data from Figure.
Jacobson 2015[33]	Unit movement from 24-bed to 30-bed unit in Dec 2013. Fall rate for 2012 and 2013 provided, but "current fall rate" in 2014 reported implying this was not a complete year. OBD unable to be estimated even though number of beds reported. Dose unable to be estimated<br><br>Incidence rate ratio for falls rate calculated using poisson command in Stata to implement an exponential-mean model, robust variance estimate used, using the monthly rates of falls and injurious falls from Figure as the inputs.<br><br>Mean difference in rate of falls estimated using regress command in Stata on monthly falls rate data from Figure.
Jeffers 2013[34]	OBDs estimated by multiplying number of beds by time.<br>Dose calculated by dividing mean number of beds monitored (reported as 12 per day) by the number of beds in the facility (525 acute beds).<br>Quarterly falls rates per 1000 OBD presented in Figure 7.<br>Number of falls estimated by multiplying falls rate by OBD/1000.<br>Note that only 3 control period measurements provided, so average of first 3 control period measure is NA.<br><br>Incidence rate ratio for falls rate and injurious falls rate calculated using poisson command in Stata to implement an exponential-mean model, robust variance estimate used, using the monthly rates of falls and injurious falls from Figure as the inputs.<br><br>Mean difference in rate of falls estimated using regress command in Stata on monthly falls rate data from Figure.
Jones 2021[35]	Data extraction for this study was complicated by a subset of patients consenting to have the intervention, falls data being presented from 13 of 15 sites, and falls data being presented per admission rather than per 1000 OBD. No average LOS data are presented that would allow a conversion to OBD. A rate ratio for falls and injurious falls was still calculated by assuming average LOS for intervention was equivalent to control.<br>A mid-bin approach for data in Table 1 was taken to estimate bed-size when a non-specific bed size for each hospital was listed. Total bed size =150+12.5+12.5+62.5+12.5+12.5+12.5+12.5+12.5+12.5+12.5+12.5+12.5+12.5+12.5<br>Occupied bed days estimated using bed size multiplied by time.<br>Dosage estimated from data presented in Table 1 using number of cameras during the intervention period as numerator and number of beds estimate as denominator.<br>Mid-bin approach taken to estimate patient age.<br><br>Incidence rate ratio for falls rate and injurious falls rate calculated using poisson command in Stata to implement an exponential-mean model, robust variance estimate used, using the monthly rates of falls and injurious falls from text as the inputs. Robust standard error created with only two data points was non-sensical. Conventional standard error used.

	Mean difference unable to be calculated due to falls data being presented as falls per admission.
Kalafus 2025[36]	Rate of falls per 1000 OBD taken from Table 4.<br>Number of falls taken from Table 4.<br>OBD estimated using these data.<br>Number of falls with injury taken from Table 4.<br>Rate of falls with injury calculated using number of falls with injury and estimate of OBD.<br>Data on proportion of patients who became fallers not presented.<br>Dose estimated by multiplying 448 patients by 13.1 days per patient of monitoring (mean), and dividing this by estimate of OBD during intervention period.<br>Time-series data values estimated using plot digitizer.<br><br>Incidence rate ratio for falls rate and falls with injury rate calculated using iri command in Stata using the number of falls and estimated number of OBDs as the inputs.<br><br>Mean difference in rate of falls estimated using iri command in Stata using the number of falls and estimated number of OBDs as the inputs.
Kolin 2010[37]	Data extraction for this study was complicated by data being presented separately for the baseline period across two figures (Figure 1- 2008, and Figure 8 – 2009). A line of best fit analysis was presented using just the Figure 8 2009 data indicating an analysis performed by the authors excluding the baseline data from 2008. We used these data for the time-based analyses.<br>Data were also for a specific site (Horizon - 2009) and for UPMC overall. The advantage of the Horizon site data is that there is a singular point of implementation. For the overall data there are two implementation points (June 2009 for paper documentation sites and September 2009 for electronic record sites). This would mean junking data from June, July and August from the overall UPMC data as it is unclear whether this is intervention or control period data. We have chosen to use the overall data as the Horizon data is a subset of this and even with the junking of Jun to Sept data, there is more falls data overall available using the UPMC-wide results.<br>The raw number of falls and falls with injury per month are reported. The number of occupied bed days could not be calculated as no indication of the number of beds was provided and no data in terms of falls per 1000 OBDs presented for the overall results.<br>Incidence rate ratio for number of falls per month and number of injurious falls per month were calculated using poisson command in Stata using the monthly number of falls and injurious falls from Figures 1 and 8 as the inputs.<br>Mean difference in fall rate could not be calculated without the relevant OBD data.<br>For the time-based analyses, we still used these data to calculate whether the relevant individual or groups of time points are higher or lower than the mean for that period by converting to a standardised effect size using the standard deviation of these data points.
Latham 2023[38]	Extracting data from this report complicated by lack of clarity as to whether the falls data reported relate just to high falls risk patients or the entire ward. The text across the report is ambiguous in this regard. The number of falls per month for each of the two wards was presented in Figure 2. By estimating the number of occupied bed days using number of beds in each ward reported in text and statement that wards are run at >90% occupancy provided in text, an estimate of number of OBDs was generated by multiplying number of beds by 0.95 to estimate OBD. This estimate was checked against the report of falls per 1000 OBD for overall sample summarized over 3-month blocks reported in Figure 1. The estimates we generated (Pre=2.17 falls per 1000 OBD,

	Post=1.83 falls per 1000 OBD) were discordant with data presented in Figure 1 (Pre=4.48 falls per 1000 OBD, Post=3.91 falls per 1000 OBD) in terms of magnitude but were close in terms of incidence rate ratio (0.84 compared to 0.87). This indicates that if the numerator data for our estimate (taken from Figure 2) and the data reported in Figure 1 were the same, then the denominators were substantially different in an absolute sense, but less so in a ratio sense. This may be explained if the OBD used to create Figure 1 were just the OBD of the high falls risk patients who had roughly half of the OBD compared to overall. However, the language in the results section and accompanying the figure does not support this hypothesis, and it may instead be a calculation error.<br><br>Data analysis complicated by presentation of data for two wards (3F and 4C) separately. We have decided to analyse the data separately for these wards as the variable percentage change in sitter was quite different for these wards and may explain different results between wards for falls outcomes. Dose data were presented in text at the study level, so were applied in the same way to both wards. Dose is based on 10 mobile video monitoring units being available across 63 beds.<br><br>We have used the fall number data presented in Figure 2 and assumed that this represents the number of falls for the whole ward. We have used our OBD estimates based on multiplying the number of beds on each ward by 0.95 based on what is reported in text.<br><br>Incidence rate ratio for falls rate calculated using poisson command in Stata to implement an exponential-mean model, robust variance estimate used, using the monthly number of falls and estimate of OBDs as exposure variable from Table 1 as inputs.<br><br>Mean difference in rate of falls estimated using regress command in Stata on number of falls data in Figure 2 and estimates of OBD as the outcome.
Melin 2018[39]	No information available to permit calculation of dose.<br>Number of falls, OBDs and falls rate per 1000 OBD taken from Table 1.<br><br>Incidence rate ratio for falls rate calculated using poisson command in Stata to implement an exponential-mean model, robust variance estimate used, using the monthly number of falls and OBDs as exposure variable from Table 1 as inputs.<br><br>Mean difference in rate of falls estimated using regress command in Stata on monthly falls rate data from Table 1.
Osborne 2023[40]	Data extraction for this study was complicated by patient time and falls wearing the sock being compared to patient time and falls not wearing the sock, within patient. There is bias introduced here as patients were not provided with the sock or taken off the sock if they were determined to not or no longer be at risk of falls.<br>Data for falls, OBD, and falls rate taken from Table 2.<br><br>Incidence rate ratio for falls rate calculated using iri command in Stata using the number of falls and number of OBDs as the inputs.<br><br>Mean difference in rate of falls estimated using iri command in Stata using the number of falls and number of OBDs as the inputs.

Overcash 2020[41]	Conference abstract reports only rate of unwitnessed falls. This was used in absence of overall fall rate.<br>Dose unable to be estimated.<br>A "50% reduction in unwitnessed fall rate (0.46 falls per 1,000 patient days)" is reported, however it is not clear whether this is the intervention or comparison rate of falls. Assumption made this is the intervention rate.<br>OBDs unable to be estimated, despite number of beds (152) being listed, the period of time involved is not specified clearly (PDSA cycles taking place over one year).<br><br>Incidence rate ratio for falls rate calculated using poisson command in Stata to implement an exponential-mean model, robust variance estimate used, using the falls rate data from text and that assumed above as the inputs. Robust standard error created with only two data points was non-sensical. Conventional standard error used.<br><br>Mean difference in rate of falls not able to be calculated.
Potter 2017[42]	Data extraction from this paper was complicated by the design and intervention. There were video-based depth sensors and conventional bed sensors. A baseline period had neither of these interventions. Phase 1 of the study had the video-based depth sensors, though it does not appear they were used for the purpose of notifying staff of a bed exit, rather "to capture video images of any actual falls occurring in patient rooms during the pilot period and to examine characteristics of those falls". During phase 2 of the study, the bed sensors were added to a medicine unit (the evaluation unit), while another medicine unit was designated as a comparison unit and did not have the bed sensors, but did have the video-based depth sensors.<br>The comparison within the evaluation unit between the baseline period (no depth or bed sensors) and phase 2 (bed and depth sensors) is what we use in our base case.<br>May benefit from a sensitivity analysis where we include also a comparison between the evaluation unit and the comparison unit within phase 2.<br>Dose not able to be precisely calculated, though likely to be close to 1 as all beds had the bed sensor added unless the patient required a "specialty bed". A value of 0.9 was estimated based on this description.<br>These two units combined had 53 beds but the allocation to each was not specified. From the text it says the evaluation unit had 14 falls during Phase 2 and a fall rate/1000 OBD of 2.22 for the evaluation unit. From this, it can be estimated that there were 6306 OBD in phase 2 for the evaluation unit. Given the period of time of phase 2 (April 2015 to January 2016 – 10 months), this would indicate the evaluation unit must have at least 21 beds. However, this could also be explained by <100% occupancy. Therefore, an assumption was made that the rate of OBD per month was consistent within the evaluation unit between the baseline and Phase 2 (base-case analysis).<br><br>Fall rates were taken from Table 1. Fall with injury rates taken from Table 2. Number of falls estimated using falls rate data and OBD data or estimates, then rounded.<br><br>Incidence rate ratio for falls rate and injurious falls rate calculated using poisson command in Stata to implement an exponential-mean model, robust variance estimate used, using the falls rate data from Table 1 and injurious falls rate data from Table 2 as the inputs. Robust standard error created with only two data points was non-sensical. Conventional standard error used.<br><br>Mean difference in rate of falls estimated using iri command in Stata on falls rate data and OBD data from text and OBD estimates.

	Note for the collection of falls data for the base case analysis, the use of the depth sensor to detect falls during phase 2 was present in addition to incident reports, but not the baseline. This may lead to a greater detection of falls in the intervention period and bias results against the intervention.
Quigley 2009[43]	Data extraction complicated by falls rate data not being presented over the same time periods as the injurious falls rate data.<br>Falls rate data presented were by unit (1 & 2) and for the final quarter in 2003 and first quarter in 2007. Injurious falls rate data presented over the entire baseline period, and the entire study period.<br>This will necessitate two separate entries in the dataset.<br>Dose not able to be calculated.<br><br>Incidence rate ratio for falls rate and injurious falls rate calculated using poisson command in Stata to implement an exponential-mean model, with data clustered by unit and robust variance estimates used, using the falls rate data from text and injurious falls rate data from Figure 1 as the inputs.<br><br>Mean difference in rate of falls estimated using regress command in Stata with data clustered by unit and robust variance estimates used, using falls rate data from text and.
Rivera 2023[44]	Data extraction complicated by presentation of multiple subtypes of falls classification. Multiple types of injury classification presented. Overall fall numbers and total injury numbers used. Note the author draws attention in text to the subcategory of injury that favours the intervention in addition to the overall falls injury category that does not.<br><br>Total number of falls, falls per 1000 OBD and number of beds on unit presented. OBD calculated using total number of falls and falls per 1000 OBD data presented.<br><br>P-values from non-parametric statistical comparisons presented in paper from using Wilcoxon Mann-Whitney tests.<br><br>Incidence rate ratio for falls rate calculated using iri command in Stata using the number of falls and number of OBDs as the inputs.<br><br>Mean difference in rate of falls estimated using iri command in Stata using the number of falls and number of OBDs as the inputs.
Rockford Memorial Hospital 2015[45]	Extraction of data from this abstract was complicated by the periods of time for the comparison of raw number of falls data (2012 vs 2014) not being the same as the injurious falls rate per 1000 OBD data (pre/post intervention commencement over an unspecified period across 2013-2014). This necessitates two entries in the dataset.<br>Dose unable to be estimated.<br><br>Incidence rate ratio for falls rate calculated using iri command in Stata using fall numbers in 2021 and 2014 reported in text, and the assumption that the OBDs over these periods were equivalent.<br><br>Incidence rate ratio for injurious falls calculated using poisson command in Stata to implement an exponential-mean model, robust variance estimate used, using injurious falls rates pre and post tele-sitter intervention reported in text. Robust standard error created with only two data points was non-sensical. Conventional standard error used.

	Mean difference in falls rate unable to be calculated.
Rosete 2015[46]	Data for stroke patients only presented.<br>Fall rate pre and post intervention presented.<br>Dose unable to be calculated.<br><br>Incidence rate ratio for falls rate calculated using poisson command in Stata to implement an exponential-mean model, robust variance estimate used, using falls rates pre and post intervention reported in text. Robust standard error created with only two data points was non-sensical. Conventional standard error used.<br><br>Mean difference in falls rate unable to be calculated.
Sand-Jecklin 2015[47]	The design of this study was a staggered entry pre-post study. Two wards commenced intervention Jan 2013, two wards July 2013. Both pairs of wards had 6 months pre and post data indicating use of a parallel control that controls for annual cyclical effects.<br>Fall numbers presented in text when broken down by unit and when combined are not concordant (30 falls units 1&2 post, 19 falls units 3&4 post, but 51 falls units 1,2,3&4 post when reported combined). The data in Table supports the broken down data, so we used this. Assumption made that OBDs evenly split between unit clusters.<br>Dose estimated by dividing number of OBDs by time (assuming 100% occupancy), and using this as the denominator, with 14 beds per unit as the numerator.<br><br>Incidence rate ratio for number of falls calculated using poisson command in Stata, using number of falls data and occupied bed days reported in text when separated by unit groups. Data clustered by unit groups and robust standard errors used.<br><br>Mean difference in falls rate calculated using regress command in Stata, using rate of falls calculated for each unit based on the raw falls numbers presented and the OBD estimated. Data clustered by unit groups and robust standard errors used.
Seow 2021[48]	Data extraction from this study complicated by patients being coded as having a fall or not during their hospital stay (yes / no). Setting was three acute wards in Singapore.<br>Actual number of fallers pre and post intervention not specified. An adjusted odds ratio and 95% CI presented for the outcome of faller vs non-faller presented within the paper was extracted.<br>Unable to calculate falls rates.<br>Unable to calculate dose.<br>Adjusted odds ratio for faller vs non-faller comparison reported in paper extracted.
Skolka 2023[49]	Extraction of data from this study is complicated by the multiple types of falls data presented, the trial design, and how falls rate data have been generated and presented. We have used data for total falls per 1000 patient-days and falls with injury per 1000 patient-days (Table 1).<br><br>Analysis of falls per 1000-patient days for this study is nuanced by the presence of 95% CIs in Table 1, drawn from hospital falls data reported quarterly for each unit (5 quarters pre, 5 quarters post). However, even with this there are insufficient datapoints to enable a meta-regression model or a mixed model analysis approach that can use the whole dataset and appropriately model the unit and period effects along with their interaction due to model overfit.

	Our base-case analysis compares the pre- and post-intervention periods within the neurology (intervention) ward. May benefit from sensitivity analyses comparing the neurology (intervention) and acute (control) wards within the post-intervention period.<br><br>Incidence rate ratio for falls rate calculated using poisson command in Stata to implement an exponential-mean model, robust variance estimate used, using falls rates pre and post intervention reported in text. Robust standard error created with only two data points was non-sensical. Conventional standard error used.<br><br>Mean difference in falls rates calculated using number of quarterly unit observations rate as reported in the text, the mean of these quarterly rate of falls observations per 1000 person-days as reported in Table 1, and a standard deviation derived from the 95% CI presented in Table 1. Here the range of the 95%CI was divided by 3.92 to yield the standard error. The standard error was multiplied by the square root of the number of quarterly observations to yield the standard deviation. The ttesti command in Stata was then implemented using these inputs.<br><br>Of note is the difference in point estimate in the potential sensitivity analysis from the base case analysis. Even though the control wards increased their number of falls going from the pre-intervention to post-intervention periods, they were still substantially lower than the intervention ward.
Sosa 2024[50]	Data extraction complicated by presentation of median (IQR) length of stay per high-risk patient rather than mean, and falls being reported as "fall during hospitalisation" rather than falls rates. Estimates for rates of falls per patient day generated by assuming one fall per patient and deriving an estimate of OBDs drawn from the reported median and IQR using formula mean=(lower quartile + 2*median + upper quartile)/4 [see X. Wan, W. Wang, J. Liu and T. Tong (2014), "Estimating the sample mean and standard deviation from the sample size, median, range and/or interquartile range", BMC Medical Research Methodology, 14: 135][51].<br><br>Mid-bin approach used to estimate age.<br><br>Incidence rate ratio for falls rate calculated using iri command in Stata using the number of falls and number of OBDs as the inputs.<br><br>Mean difference in rate of falls estimated using iri command in Stata using the number of falls and number of OBDs as the inputs.
Spetz 2007[52]	Parallel control study comparing rates of falls from patients in 10 rooms with video monitoring within a ward with rates from patients in 14 other rooms within the same ward. Falls number, average length of stay, and number of patients data from Table 1 used.<br><br>Incidence rate ratio for falls rate calculated using iri command in Stata using the number of falls and number of OBDs as the inputs.<br><br>Mean difference in rate of falls estimated using iri command in Stata using the number of falls and number of OBDs as the inputs.
Vassallo 2004[53]	Parallel control group study at the ward level.<br>Dose not able to be calculated. No detailed description, however, notes provided that use of alarms on control wards was likely to be suboptimal as "there is no evidence that

	it was implemented in its entirety, nor was there any regular review of the parts' effectiveness".<br><br>Table 2 provided relevant data. Note, the authors in the discussion claim a 15% reduction in falls, yet the falls per 1000 OBD is higher in the intervention group. A Mann-Whitney U test was used to compare falls per 1000 OBD between groups which would have concealed some participants with very high numbers of falls.<br><br>Incidence rate ratio for falls rate calculated using iri command in Stata using the number of falls and number of OBDs as the inputs.<br><br>Mean difference in rate of falls estimated using iri command in Stata using the number of falls and number of OBDs as the inputs.<br><br>Odds ratio presented in Table 3 used for fallers vs non-fallers analysis.
Visvanathan 2022[54]	Actual number of falls and injuries not presented for each group, only the number of fallers by group, and the total number of falls and injuries in the study overall (not by group).<br><br>Incidence rate ratios and odds ratios presented in the paper were used.<br><br>Mean difference in falls rate was calculated using iri command in Stata, using estimates of number of falls and OBDs as inputs. Values for OBD were estimated using the formula mean=(lower quartile + 2*median + upper quartile)/4 [see X. Wan, W. Wang, J. Liu and T. Tong (2014), "Estimating the sample mean and standard deviation from the sample size, median, range and/or interquartile range", BMC Medical Research Methodology, 14: 135] [51] using data in Table 1 for the median and IQR but data from Table 2 for the n. Number of falls was estimated by using the falls rates per 1000 days and multiplying this by the estimate of OBDs, dividing by 1000, then rounding.<br><br>The standard error of this mean difference in rate of falls calculation needed to be inflated for a design effect.[55] No ICC value was reported in this paper so the ICC of 0.014 from Cumming et al 2008 was used. There were data from 3114 patients in this study across 3 wards, making the average n per ward = 1038. The Design Effect = 1+(n-1)**p*.<br>The Design Effect = 1+ (1038-1)*0.014 = 3.31875.<br>We then calculated the normal SE using iri and multiplied this by the square root of the Design Effect.<br>The conventional iri incidence rate difference standard error is 0.875638<br>The new cluster standard error of the mean difference=0.875638*SQRT(15,518)=3.4493<br><br>Note is made that this inflated standard error is likely to produce an overly large standard error as stepped wedge trials are generally more powerful than equivalent parallel cluster trials.[56]
Vortuba 2016[57]	Number of falls presented Table 1.<br>Dose unable to be calculated as number of beds in the 3 units not presented. Numerator data available (up to 12 patients monitored) but not denominator.<br>Number of beds and occupied bed days not presented.<br>Nine months of pre-intervention data were compared to 9 months of post-intervention data. Assumption of equivalent length of stay made when calculating incidence rate ratios.

	Incidence rate ratio for falls rate calculated using iri command in Stata using the number of falls and number of OBDs as the inputs.<br><br>Mean difference in falls rates unable to be calculated.
Weppner 2024[58]	Data extraction complicated by reporting of monthy incidence of falls from chair. This was used as total falls was not presented. Rate of falls/1000 OBD and number of falls presented in text. OBDs estimated using these data.<br><br>Incidence rate ratio for falls rate calculated using iri command in Stata using the number of falls and estimated number of OBDs as the inputs.<br><br>Mean difference in rate of falls estimated using iri command in Stata using the number of falls and estimated number of OBDs as the inputs.<br><br>Odds ratio calculated using csi command in Stata using data presented in text.<br><br>Time-series data taken from figure 3.
Westle 2017[59]	Data extraction for this study complicated by presentation of the results for the main pilot ward (neuro), with detailed extrapolation of fall rate and injury rate data (Figure 4), then presentation of data for two additional wards (Table 1). The dosage estimates were not clear at the ward level but were stated as being 21 cameras for 3 wards each with ~32 beds.<br>We have included all three wards in the dataset for the falls rate and injury rate analysis, but used the data in figure 4 for time-based analyses.<br>Data for percentage change in sitter use not presented, rather a hypothetical statement that 60FTE would have been needed to cover the patient monitoring completed by the video system. A value of zero was used here for this variable.<br><br>Time-based analyses:<br>Data for fall rates per 1000 OBD presented in Table 1 used. Figure 4. Data in figure 4 converted to numeric using PlotDigitizer.<br><br>Overall analyses:<br>Each ward has 34 beds so OBD estimated by multiplying this by time assuming 100% occupancy. Number of falls estimated using OBD and falls rates estimates.<br><br>Incidence rate ratio for falls rate and injury rate calculated using poisson command in Stata to implement an exponential mean model, using the rate data presented in Table 1 as input. Robust variance used with data clustered by ward.<br><br>Mean difference in rate of falls calculated using regress command in Stata, using rate of falls data presented in Table 1 as input. Robust variance used with data clustered by ward.
Woltsche 2022[60]	No data on use of bed alarms provided for the control or intervention condition, only video monitoring for the intervention being 6 video monitors per ward and zero for control. Dose data complicated by this. However, specification of 6 cameras per ward (18 total) was specified along with ward size (total=77 beds), yielding a dose of 0.23.

	Data for total falls and OBDs (Table 1) was used for our analyses. It is noted that the description of total falls rate in text does not concord with falls rates calculated from data in table.<br><br>Data presented by ward for each of 3 wards pre and post implementation of the video monitoring intervention. This permitted use of a linear mixed model analysis approach.<br><br>Incidence rate ratio for falls rate per 1000 OBD calculated using mepoisson command in Stata using the number of falls as the outcome, number of OBDs as the exposure variable, and a random intercept for ward.<br><br>Mean difference in rate of falls calculated using meglm command in Stata using the rate of falls calculated from data in Table 1 as the outcome, and a random intercept for ward.
Wong-Shee 2013 (paper plus thesis)[61,62]	Extraction of data from this study complicated by the A-B-A design. We have only considered the first A-B part of this design due to the attrition with the second A phase. These data are within participant similar to Osborne 2023.[40]<br>Number of falls overall and occupied bed days taken from PhD thesis.<br>Injury data taken from PhD thesis.<br>Incidence rate ratio for falls rate presented in Table 1 used.<br>Injury rate ratio and mean difference in fall rates calculated using iri command in Stata using number of injurious falls or number of falls, and number of occupied bed days reported as inputs.<br>We note the standard errors calculated this way are likely to be too large given they do not take into account the within subject nature of the study design.
Wright 2022[63]	Extraction of data from this study complicated by presentation only of night-time falls. These have been used in the absence of total falls data. Also, complicated by the baseline line on Figures 2 and 3 being in different locations. The text indicates the line should be to the right of the Feb 2018 marker in Figure 3 as it is in Figure 2. We have made this assumption based on the text and Figure 2.<br>No mention is made of use of other forms of alarm during the control period, so dose is assumed to be zero at baseline. Quarterly fall rates per 1000 OBD from Figure 3 used and converted to numeric using PlotDigitizer. Data from Figure 2 used to calculate percentage OBDs and converted to numeric using PlotDigitizer, then multiplied by 24 beds to get actual OBDs. Falls numbers estimated using these figures then rounding.<br><br>Incidence rate ratio for falls rate per 1000 OBD calculated using poisson command in Stata to implement an exponential-mean model, robust variance estimate used, using falls rates pre and post intervention reported in Figure 3.<br><br>Mean difference in falls rate calculated using regress command in Stata, using rate of falls pre and post intervention reported in Figure 3.

References:

1) Barker AL, Morello RT, Wolfe R, Brand CA, Haines TP, Hill KD, Brauer SG, Botti M, Cumming RG, Livingston PM, Sherrington C. 6-PACK programme to decrease fall injuries in acute hospitals: cluster randomised controlled trial. BMJ. 2016 Jan 26;352.
2) PlotDigitizer website. Porbital 2026, https://plotdigitizer.com/.
3) Stata SE version 18.0. StataCorp College Station 2023, Texas.

4) Cozart HC. Environmental effects on incidence of falls in the hospitalized elderly. Texas Woman's University; 2009.
5) Cumming RG, Sherrington C, Lord SR, Simpson JM, Vogler C, Cameron ID, Naganathan V. Cluster randomised trial of a targeted multifactorial intervention to prevent falls among older people in hospital. Bmj. 2008 Apr 3;336(7647):758-60.
6) Hardin SR, Dienemann J, Rudisill P, Mills KK. Inpatient fall prevention: use of in-room webcams. Journal of patient safety. 2013 Mar 1;9(1):29-35.
7) Kwok T, Mok F, Chien WT, Tam E. Does access to bed-chair pressure sensors reduce physical restraint use in the rehabilitative care setting?. Journal of clinical nursing. 2006 May;15(5):581-7.
8) Pu D, Stephen K, McDonald C, Baker J, Sinforosa G, Hutchinson A, Bradley K, Woods-Lyon K, Tuck M, Brusco N, O'brien L. Investigating the effectiveness of mobilization alarms to prevent hospital falls using disinvestment: A randomised clinical trial. International journal of nursing studies. 2026 Jan 23:105320.
9) Sahota O, Drummond A, Kendrick D, Grainge MJ, Vass C, Sach T, Gladman J, Avis M. REFINE (REducing Falls in In-patieNt Elderly) using bed and bedside chair pressure sensors linked to radio-pagers in acute hospital care: a randomised controlled trial. Age and ageing. 2014 Mar 1;43(2):247-53.
10) Shorr RI, Chandler AM, Mion LC, Waters TM, Liu M, Daniels MJ, Kessler LA, Miller ST. Effects of an intervention to increase bed alarm use to prevent falls in hospitalized patients: a cluster randomized trial. Annals of internal medicine. 2012 Nov 20;157(10):692-9.
11) Tideiksaar R, Feiner CF, Maby J. Falls prevention: the efficacy of a bed alarm system in an acute-care setting. The Mount Sinai Journal of Medicine, New York. 1993 Nov 1;60(6):522-7.
12) Wolf KH, Hetzer K, Zu Schwabedissen HM, Wiese B, Marschollek M. Development and pilot study of a bed-exit alarm based on a body-worn accelerometer. Zeitschrift für Gerontologie und Geriatrie. 2013 Dec;46(8):727-33.
13) Baitar A, Deckers L, Messens E, Schrijvers D. Fall prevention in a palliative care unit: the use of a bed-exit alarm in practice. Palliative Medicine. 2012 Jun;26(4):471-2.
14) Bardall ME. *The implementation of call bells and exit alarms on a psychiatric unit* (Doctoral dissertation, Rutgers University-School of Nursing-RBHS), 2020.
15) Adlington K, Brown J, Ralph L, Clarke A, Bhoyroo T, Henderson M, et al. Better care: reducing length of stay and bed occupancy on an older adult psychiatric ward. BMJ Open Quality. 2018;7:e000149. https://doi.org/10.1136/bmjoq-2017-000149
16) Barker A, Kamar J, Morton A, Berlowitz D. Bridging the gap between research and practice: review of a targeted hospital inpatient fall prevention programme. BMJ Quality & Safety. 2009 Dec 1;18(6):467-72.
17) Blackburn R, Johnston P, Faultus K, Tomczak N, Jewell J, Doyle K, De la Cruz B, Jacob R, Rowe T. Preventing Fall Related Injuries In Hematopoeitic Stem Cell Transplant Patients: An Evidence Based Approach. Biology of Blood and Marrow Transplantation. 2010 Feb 1;16(2):S193.
18) Brusco, N.K., Hutchinson, A.M., Mitchell, D., Jellett, J., Boyd, L., Webb-St Mart, M., Raymond, M., Clayton, D., Farley, A., Botti, M., Steen, K., et al. 2021. Mobilization alarm triggers, response times and utilisation before and after the introduction of policy for alarm reduction or elimination: A descriptive and comparative analysis. *International journal of nursing studies*, *117*, p.103769.
19) Burtson PL, Vento L. Sitter reduction through mobile video monitoring: a nurse-driven sitter protocol and administrative oversight. JONA: The Journal of Nursing Administration. 2015 Jul 1;45(7/8):363-9.
20) Capan K, Lynch B. A hospital fall assessment and intervention project. JCOM- Journal of Clinical Outcomes Management. 2007;14(3):155-160.
21) Cook NS, Komansky BJ, Urton MS. Do no harm: a multifactorial approach to preventing emergency department falls—a quality improvement project. Journal of emergency nursing. 2020 Sep 1;46(5):666-74.
22) Cournan M, Fusco-Gessick B, Wright L. Improving patient safety through video monitoring. Rehabilitation Nursing. 2016 Oct. **doi.org/10.1002/rnj.308**

23) Cruse YC. *The Effectiveness of Continuous Video Monitoring in Reducing Inpatient Fall Rates* (Doctoral dissertation, University of Missouri-Saint Louis), 2022.
24) Cuttler SJ, Barr-Walker J, Cuttler L. Reducing medical-surgical inpatient falls and injuries with videos, icons and alarms. BMJ open quality. 2017 Oct 26;6:e000119. doi:10.1136/ bmjoq-2017-000119
25) Dadabhoy FZ, Lautar A, Schlaudecker J. Impact of Fall Reduction Interventions added to Interprofessional Bedside Rounds on an ACE Unit. Journal of the American Geriatrics Society 2017 may 1 (vol. 65), pp. s102.
26) Ergai A, Spiva L, Thurman S, Hatfield M, McCollum M, Holmes M. The effectiveness of remote video monitoring on fall prevention and nurses' acceptance. Journal of Nursing Care Quality. 2024 Jan 1;39(1):24-30.
27) Fonda D, Cook J, Sandler V, Bailey M. Sustained reduction in serious fall-related injuries in older people in hospital. Medical Journal of Australia. 2006 Apr;184(8):379-82.
28) Gervasi C, Perego E, Galli F, Torri V, Castoldi M, Bombardieri E. Prevention of falls in hospitalized patients – evaluation of the effectiveness of a monitoring system (Verso Vision) developed with artificial intelligence. Frontiers in Digital Health 2025 April 16; 7: doi.org/10.3389/fdgth.2025.1548209
29) Ha SH, Kim M, Kim H, No B, Go A, Choi M, Lee S, Ahn Y. Cancer-oriented comprehensive nursing services in Republic of Korea: Lessons from an oncologist's perspective. Medicina. 2023 Jan 11;59(1):144.
30) Halgren A, Larche J, Vizvarie M. Preventing Falls Utilizing Remote Patient Observation. Archives of Physical Medicine and Rehabilitation. 2025 Apr 1;106(4):e52-3.
31) Hatler C. How intelligent are smart beds?. Nursing Management. 2008 Feb 1;39(2):20-26.
32) Hogan-Quigley B, Renz SM, Bradway C. Fall prevention and injury reduction utilizing virtual sitters in hospitalized patients: a literature review. CIN: Computers, Informatics, Nursing. 2021 Dec 1;39(12):929-34.
33) Jacobson AK, O'Brien B, Johnson C. Falls in BMT patients. Biology of Blood and Marrow Transplantation. 2015 Feb 1;21(2):S384.
34) Jeffers S, Herring C, Searcey P, Lester K, Boyle K, Goetz-Smith H, Nelson P. Centralized video monitoring for patient Safety: A Denver Health Lean Journey. Nursing Economic$. 2013 Nov/Dec; 31(6): 298-307.
35) Jones L, Hessler K, Winter B, Kupzyk K. Use of toilet alarms in inpatient settings. Nursing2025. 2021 Jan 1;51(1):65-9.
36) Kalafus L, Hrdlicka HC, Lombardi J, Proctor S, Napolitano J, Morrill N. Implementation of a Continuous Video Monitoring Program to Decrease Inpatient Falls in a Long-Term Acute Care Hospital Setting: A Prospective Observational Cohort Study. AJN The American Journal of Nursing. 2025 Dec 1;125(12):20-7.
37) Kolin MM, Minnier T, Hale KM, Martin SC, Thompson LE. Fall initiatives: redesigning best practice. JONA: The Journal of Nursing Administration. 2010 Sep 1;40(9):384-91.
38) Latham KW. The Impact of Remote Video Monitoring on Costs and Patient Outcomes. The George Washington University. 2023.
39) Melin CM. Reducing falls in the inpatient hospital setting. JBI Evidence Implementation. 2018 Mar 1;16(1):25-31.
40) Osborne TF, Veigulis ZP, Arreola DM, Vrublevskiy I, Suarez P, Curtin C, Schalch E, Cabot RC, Gant-Curtis A. Assessment of a wearable fall prevention system at a veterans health administration hospital. Digital health. 2023 Jul;9:20552076231187727.
41) Overcash S. Improving Patient Safety In The Rehab Setting With Efficient Patient Monitoring Techniques and Other Interventions. Archives of Physical Medicine and Rehabilitation. 2020 Nov 1;101(11):e50. Research poster 1358713.
42) Potter P, Allen K, Costantinou E, Klinkenberg WD, Malen J, Norris T, O'Connor E, Roney W, Tymkew HH, Wolf L. Evaluation of sensor technology to detect fall risk and prevent falls in acute care. The Joint Commission Journal on quality and patient safety. 2017 Aug 1;43(8):414-21.

43) Quigley PA, Hahm B, Collazo S, Gibson W, Janzen S, Powell-Cope G, Rice F, Sarduy I, Tyndall K, White SV. Reducing serious injury from falls in two veterans' hospital medical-surgical units. Journal of nursing care quality. 2009 Jan 1;24(1):33-41.
44) Rivera DF, Morelli JD, Barrett-Fajardo N, Mattia A, McDonough K, Astorino V, Fox C, Wang M, Rutledge J. Transforming the paradigm of care to reduce falls on a medical-surgical unit using artificial intelligence. Journal of Informatics Nursing. 2023;8(3):18-28.
45) Rockford Memorial Hospital. Hospitals report success with video monitoring. Healthcare Risk Management 2015; 37(7): 1987263158
46) Rosete J, Neil W, Button J, Buccigrossi D, Forde K, Mallett K. Abstract NS9: A Successful Fall Prevention Program In A Mixed Nursing Unit Does Not Translate To Decreased Fall Rates For Stroke Patients. Stroke. 2015 Feb;46(suppl_1):ANS9-.
47) Sand-Jecklin K, Johnson J, Tringhese A, Daniels C, White F. Video monitoring for fall prevention and patient safety: process evaluation and improvement. Journal of nursing care quality. 2019 Apr 1;34(2):145-50.
48) Seow JP, Chua TL, Aloweni F, Lim SH, Ang SY. Effectiveness of an integrated three-mode bed exit alarm system in reducing inpatient falls within an acute care setting. Japan Journal of Nursing Science. 2022 Jan;19(1):e12446.
49) Skolka MP, Neth BJ, Brown A, Steel SJ, Hacker K, Arnold C, Toledano M, Mustafa R. Improving neurology inpatient fall rate: Effect of a collaborative Interdisciplinary Quality Improvement Initiative. Mayo Clinic Proceedings: Innovations, Quality & Outcomes. 2023 Aug 1;7(4):267-75.
50) Sosa MA, Soares M, Patel S, Trujillo K, Ashley D, Smith E, Shukla B, Parekh D, Ferreira T, Gershengorn HB. The impact of adding a 2-way video monitoring system on falls and costs for high-risk inpatients. Journal of patient safety. 2024 Apr 1;20(3):186-91.
51) Wan, W. Wang, J. Liu and T. Tong. Estimating the sample mean and standard deviation from the sample size, median, range and/or interquartile range. BMC Medical Research Methodology. 2014;14:135.
52) Spetz J, Jacobs J, Hatler C. Cost effectiveness of a medical vigilance system to reduce patient falls. Nursing economic$. 2007 Nov 1;25(6).
53) Vassallo M, Vignaraja R, Sharma JC, Hallam H, Binns K, Briggs R, Ross I, Allen S. The effect of changing practice on fall prevention in a rehabilitative hospital: the Hospital Injury Prevention Study. Journal of the American Geriatrics Society. 2004 Mar;52(3):335-9.
54) Visvanathan, R., Ranasinghe, D.C., Lange, K., Wilson, A., Dollard, J., Boyle, E., Jones, K., Chesser, M., Ingram, K., Hoskins, S. and Pham, C., 2022. Effectiveness of the wearable sensor-based ambient intelligent geriatric management (AmbIGeM) system in preventing falls in older people in hospitals. *The Journals of Gerontology: Series A*, *77*(1), pp.155-163.
55) Higgins JP, Eldridge S, Li T. Chapter 23: Including variants on randomized trials (last updated October 2019). In: Higgins JP, Thomas J, Chandler J, Cumpston M, Li T, Page MJ, et al, editor(s). Cochrane Handbook for Systematic Reviews of Interventions version 6.5. Cochrane, 2024. Available from cochrane.org/handbook.
56) Sarkodie SK, Wason JM, Grayling MJ. A hybrid approach to comparing parallel-group and stepped-wedge cluster-randomized trials with a continuous primary outcome when there is uncertainty in the intra-cluster correlation. Clinical Trials. 2023;20(1):59-70.
57) Votruba L, Graham B, Wisinski J, Syed A. Video Monitoring to Reduce Falls And Patient Companion Costs For Adult Inpatients. Nursing Economic$. 2016 Jul 1;34(4).
58) Weppner J, Gee A, Mesina K. Enhancing safety in the ischaemic and haemorrhagic stroke population: exploring the efficacy of self-releasing chair alarm belts. BMJ Open Quality. 2024 Feb 20;13(1).
59) Westle MB, Burkert GR, Paulus RA. Reducing inpatient falls and injury rates by integrating new technology with workflow redesign. NEJM Catalyst. 2017 July 10 & 2019 May 23;5(3).
60) Woltsche R, Mullan L, Wynter K, Rasmussen B. Preventing patient falls overnight using video monitoring: a clinical evaluation. International Journal of Environmental Research and Public Health. 2022 Oct 22;19(21):13735.

61) Wong-Shee A, Phillips B, Hill K, Dodd K. Feasibility, acceptability, and effectiveness of an electronic sensor bed/chair alarm in reducing falls in patients with cognitive impairment in a subacute ward. Journal of nursing care quality. 2014 Jul 1;29(3):253-62.
62) Wong Shee A. *Fall risk assessment and minimisation in a sub-acute setting* (Doctoral dissertation, La Trobe), 2013.
63) Wright K, Singh S. Reducing falls in dementia inpatients using vision-based technology. Journal of Patient Safety. 2022 Apr 1;18(3):177-81.

Supplementary Material S6: Risk of bias assessment

Risk of bias ratings for RCTs with randomization of individuals.

Risk of bias domains

Study	D1	D2	D3	D4	D5	Overall
Cozart, 2009 (pilot)	X	+	+	X	X	X
Cozart, 2009 (main trial)	X	+	+	X	X	X
Kwok et al., 2006	+	+	+	X	X	X
Sahota et al., 2014	+	+	+	X	+	X
Tideiksaar et al., 1993	-	X	X	X	X	X
Wolf et al., 2013	X	X	X	X	X	X

Domains:
D1: Bias arising from the randomization process.
D2: Bias due to deviations from intended intervention.
D3: Bias due to missing outcome data.
D4: Bias in measurement of the outcome.
D5: Bias in selection of the reported result.

Judgement
X High
- Some concerns
+ Low

Risk of bias ratings for cluster randomized controlled trials.

Risk of bias domains

Study	D1	D1b	D2	D3	D4	D5	Overall
Barker et al., 2016	+	+	+	+	+	+	+
Cumming et al., 2008	+	+	+	+	+	-	-
Hardin et al., 2013	-	X	+	+	X	X	X
Pu et al., 2026	+	-	+	+	+	+	-
Shorr et al., 2012	+	+	+	+	+	+	+

Domains:
D1 : Bias arising from the randomization process.
D1b: Bias arising from the timing of identification and recruitment of Individual participants in relation to timing of randomization.
D2 : Bias due to deviations from intended intervention.
D3 : Bias due to missing outcome data.
D4 : Bias in measurement of the outcome.
D5 : Bias in selection of the reported result.

Judgement
X High
- Some concerns
+ Low

Risk of bias ratings for non-RCTs.

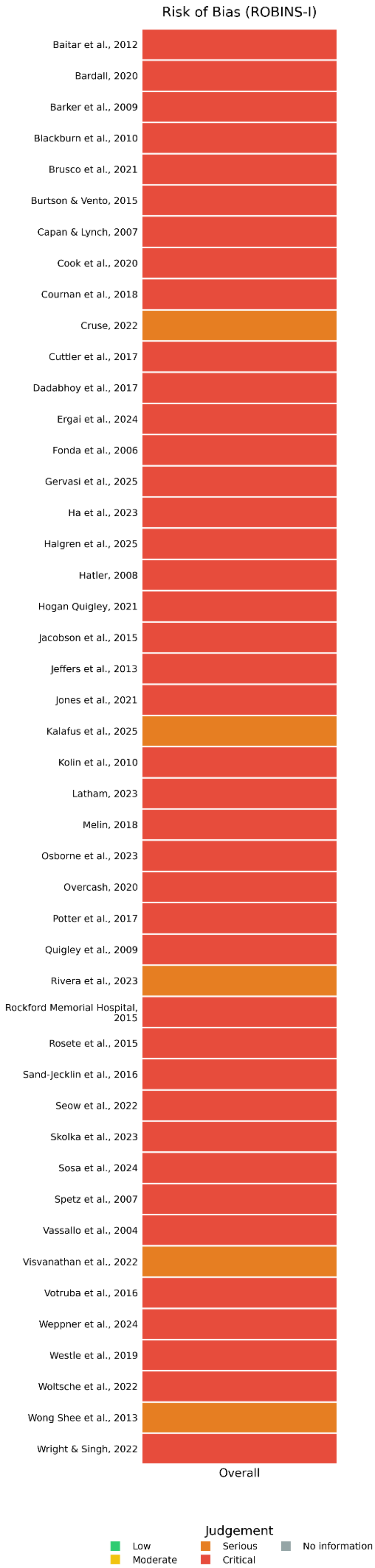

Supplementary Material S7: Subgroup analyses excluding studies with mean age <60 years.

Two studies (Bardall 2009, Barker 2009) had a mean age <60 years. Both of these studies were non-RCTs. Neither study had incidence rate ratio date for the falls with injury / 1000 OBD outcome. Subgroup meta-analyses inclusive of all trial designs for the remaining three falls outcomes have been reported below.

	Incidence rate ratio for rate of falls / 1000 OBD	Mean difference in rate of falls / 1000 OBD	Odds ratio of becoming a faller
Pooled estimate (95%CI) overall	0.80 (0.74 to 0.87)	-0.98 (-1.36 to -0.59)	0.86 (0.60 to 1.23)
n (comparisons), tau^2, I^2 overall	60, 0.05, 71.01%	43, 0.68, 63.39%	10, 0.17, 73.03%
Pooled estimate (95%CI) mean age <60 excluded	0.79 (0.73 to 0.86)	-1.04 (-1.41 to -0.67)	0.85 (0.58 to 1.25)
n (comparisons), tau^2, I^2 mean age<60 excluded	58, 0.04, 63.42%	42, 0.54, 55.88%	9, 0.19, 77.04%
Pooled estimate (95%CI) only those with mean age <60	1.20 (1.11 to 1.30)	0.61 (0.00 to 1.22)	1.07 (0.27 to 4.22)
n (comparisons), tau^2, I^2 only those with mean age <60	2, 0.00, 0.00%	1, 0.00, not calculated	1, 0.00, not calculated

Supplementary Material S8: Funnel plots across study outcomes by subgroup of whether trial is an RCT or non-RCT.

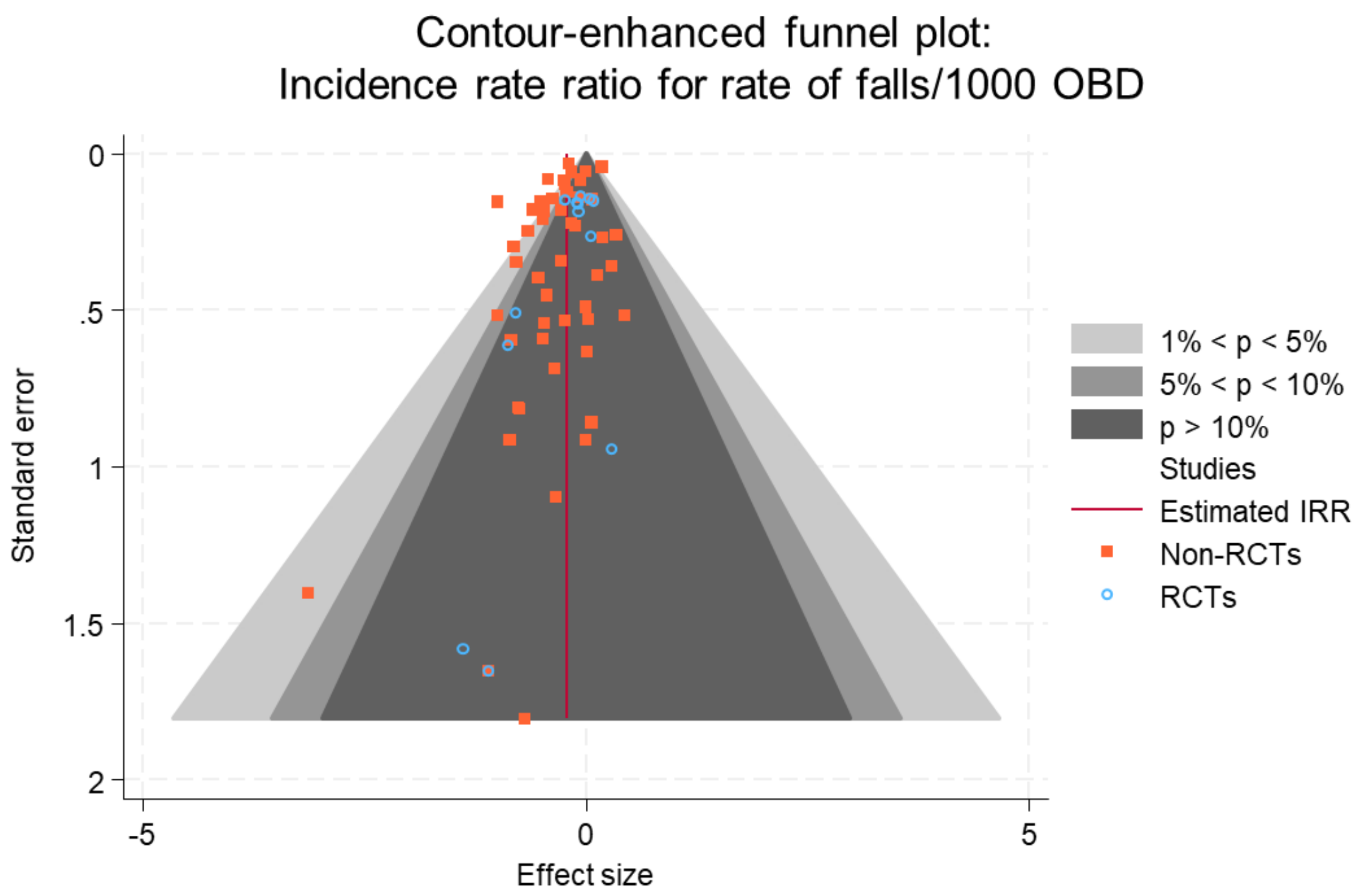

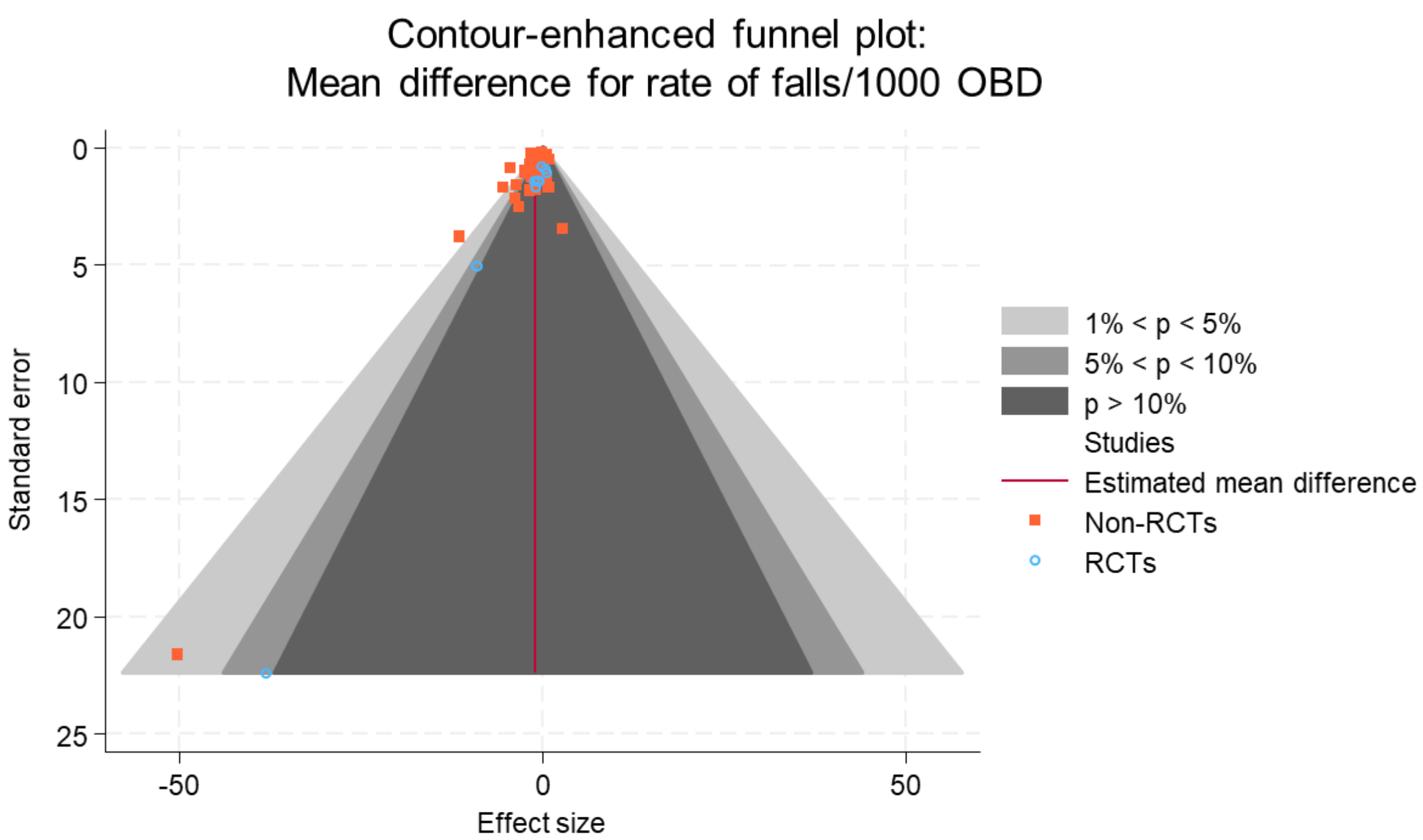

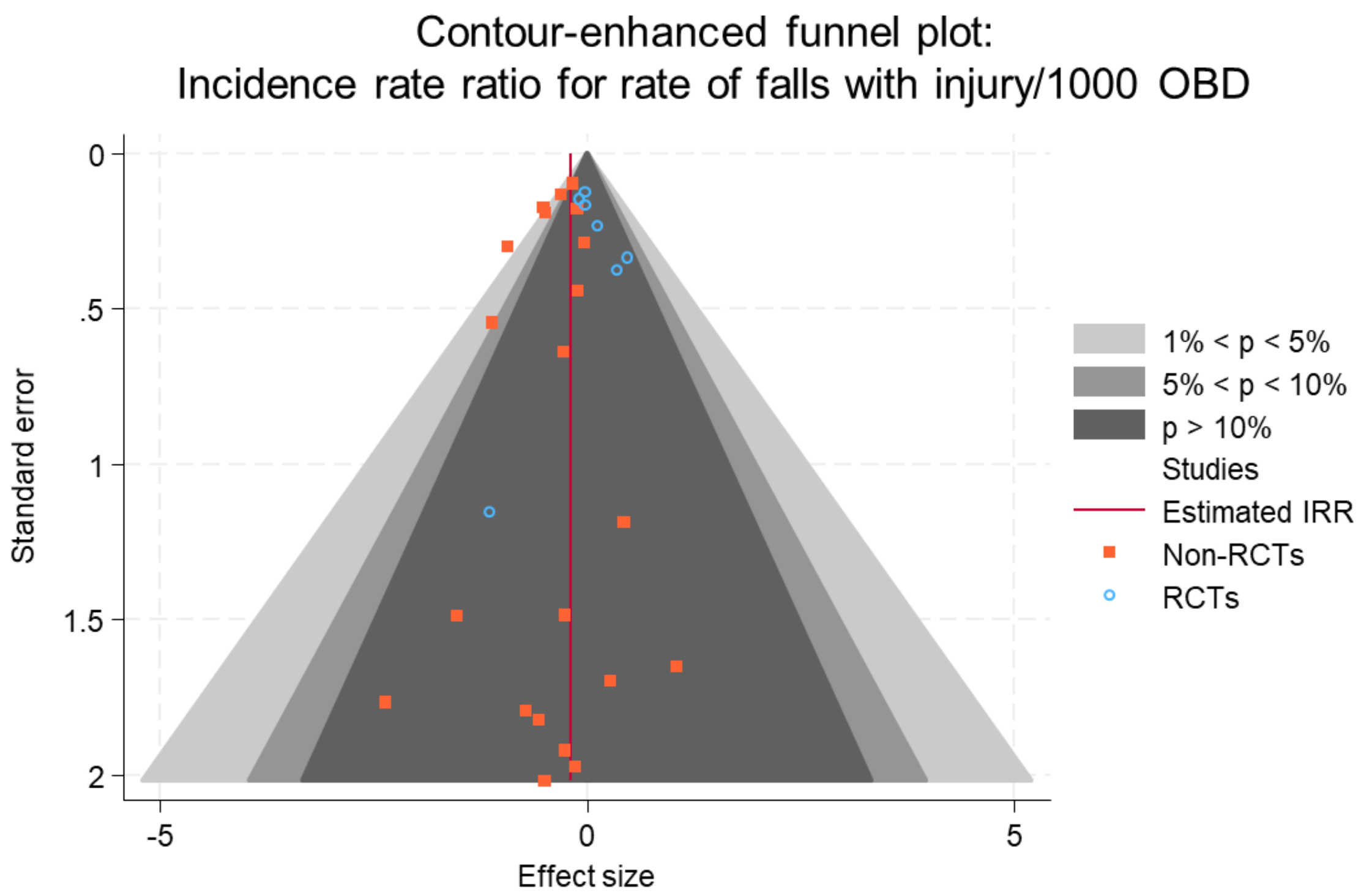
Contour-enhanced funnel plot:
Incidence rate ratio for rate of falls with injury/1000 OBD
Standard error
0
.5
1
1.5
2
-5
0
5
Effect size
1% < p < 5%
5% < p < 10%
p > 10%
Studies
Estimated IRR
Non-RCTs
RCTs

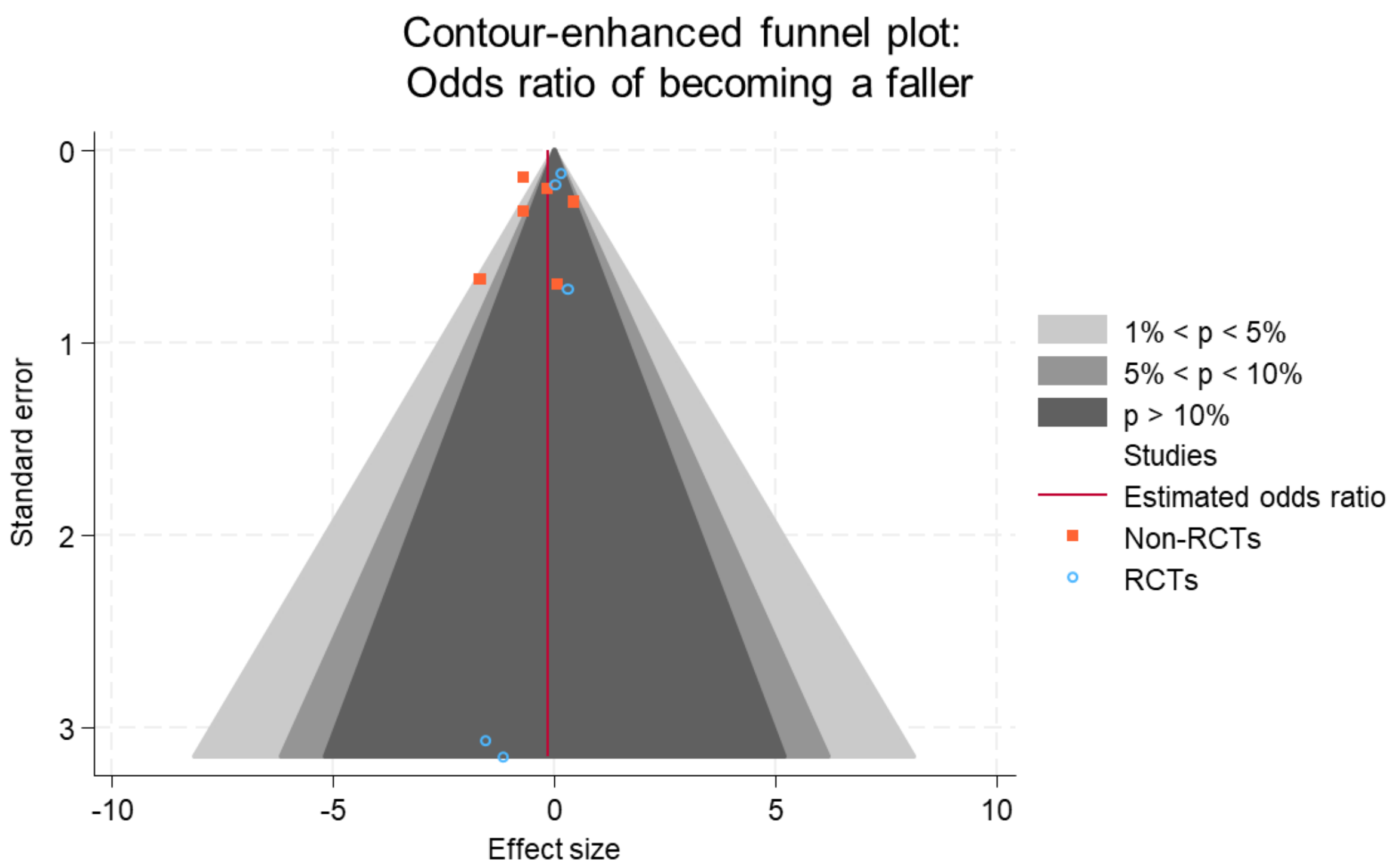
Contour-enhanced funnel plot:
Odds ratio of becoming a faller
Standard error
0
1
2
3
-10
-5
0
5
10
Effect size
1% < p < 5%
5% < p < 10%
p > 10%
Studies
Estimated odds ratio
Non-RCTs
RCTs

Supplementary Material S9: Dose-response curve for all studies.

1) IRR for rate of falls, restricted cubic splines, knots at 0.06, 0.08, 0.12 & 1.

```
One-stage random-effect dose-response model      Number of studies =         33
Optimization   = reml                                Number of obs =         34
          AIC = 18.19                                Model chi2(3) =      24.70
Log likelihood = -.09507635                           Prob > chi2 =     0.0000
------------------------------------------------------------------------------
beta_falls~r |     exb(b)   Std. err.      z    P>|z|     [95% conf. interval]
-------------+----------------------------------------------------------------
      doses1 |   .1431017   .1179887    -2.36   0.018     .0284329    .7202259
      doses2 |   3.3e+202   1.1e+205     1.35   0.178     8.45e-93           .
      doses3 |   4.1e-306   2.2e-303    -1.34   0.182            0    3.8e+142
------------------------------------------------------------------------------
------------------------------------------------------------------------------
  Random-effects parameters  |   Estimate
-----------------------------+---------------
var(doses1,doses1)           |    .4914693
var(doses2,doses2)           |     .578545
var(doses3,doses3)           |    23.97178
cov(doses1,doses2)           |   -.4676762
cov(doses1,doses3)           |   -3.405005
cov(doses2,doses3)           |    3.014572
--------------------------------------------
LR test vs. no random-effects model = 5.2096109       Prob >= chi2(6) = 0.5172
```

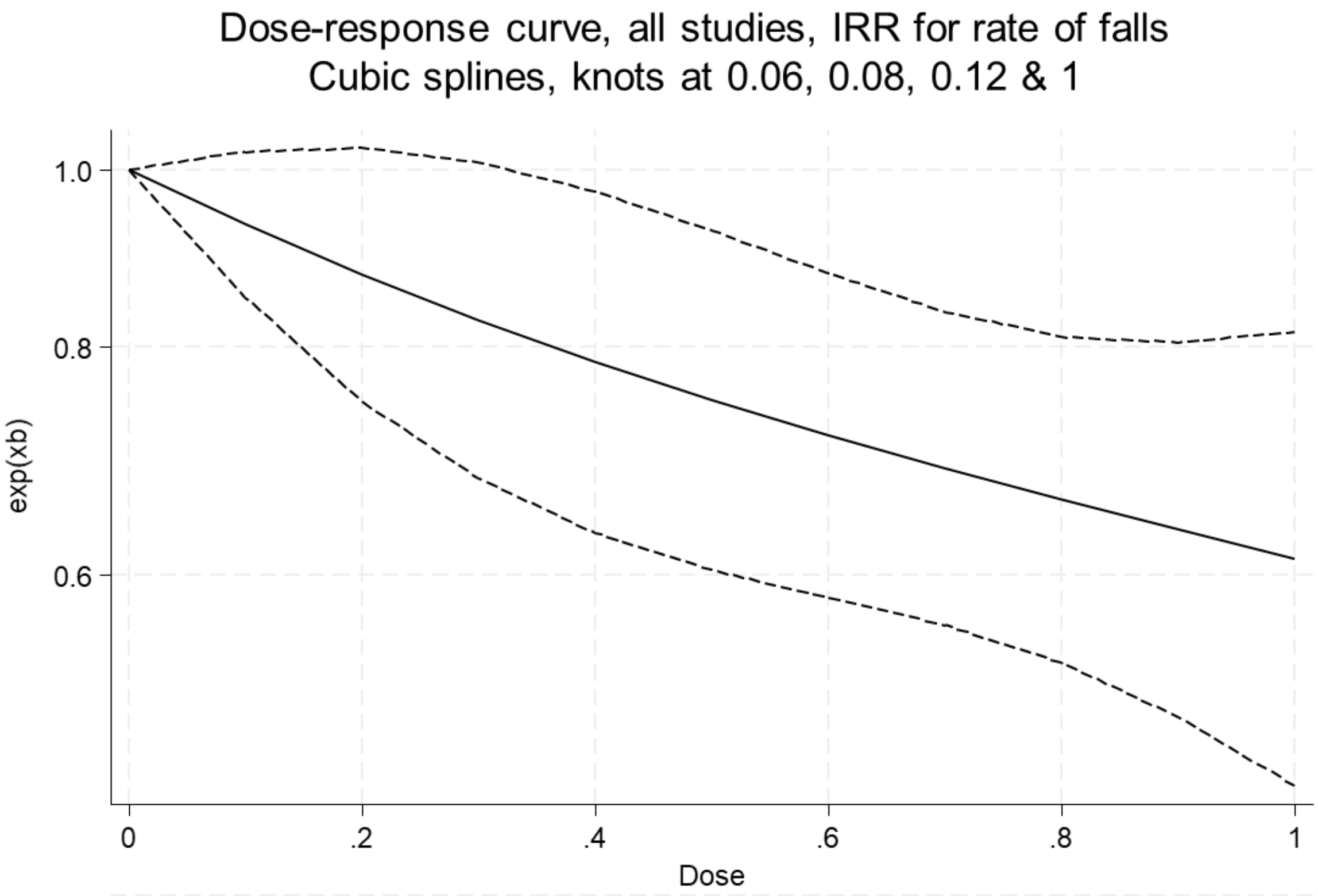

2) Mean difference in rate of falls, restricted cubic splines, knots at 0.06, 0.08 & 1.

```
One-stage random-effect dose-response model      Number of studies =        16
Optimization   = reml                                Number of obs =        17
          AIC = 47.54                                Model chi2(2) =     14.36
Log likelihood = -18.771513                            Prob > chi2 =    0.0008
------------------------------------------------------------------------------
          md | Coefficient  Std. err.      z    P>|z|     [95% conf. interval]
-------------+----------------------------------------------------------------
      doses1 |   -4.08396   2.800219    -1.46   0.145    -9.572289    1.404368
      doses2 |   9.833712   80.33349     0.12   0.903     -147.617    167.2845
------------------------------------------------------------------------------
---------------------------------------------
  Random-effects parameters  |   Estimate
-----------------------------+---------------
var(doses1,doses1)           |    36.29168
var(doses2,doses2)           |    23570.53
cov(doses1,doses2)           |   -909.1333
---------------------------------------------
LR test vs. no random-effects model = 5.547326        Prob >= chi2(3) = 0.1358
```

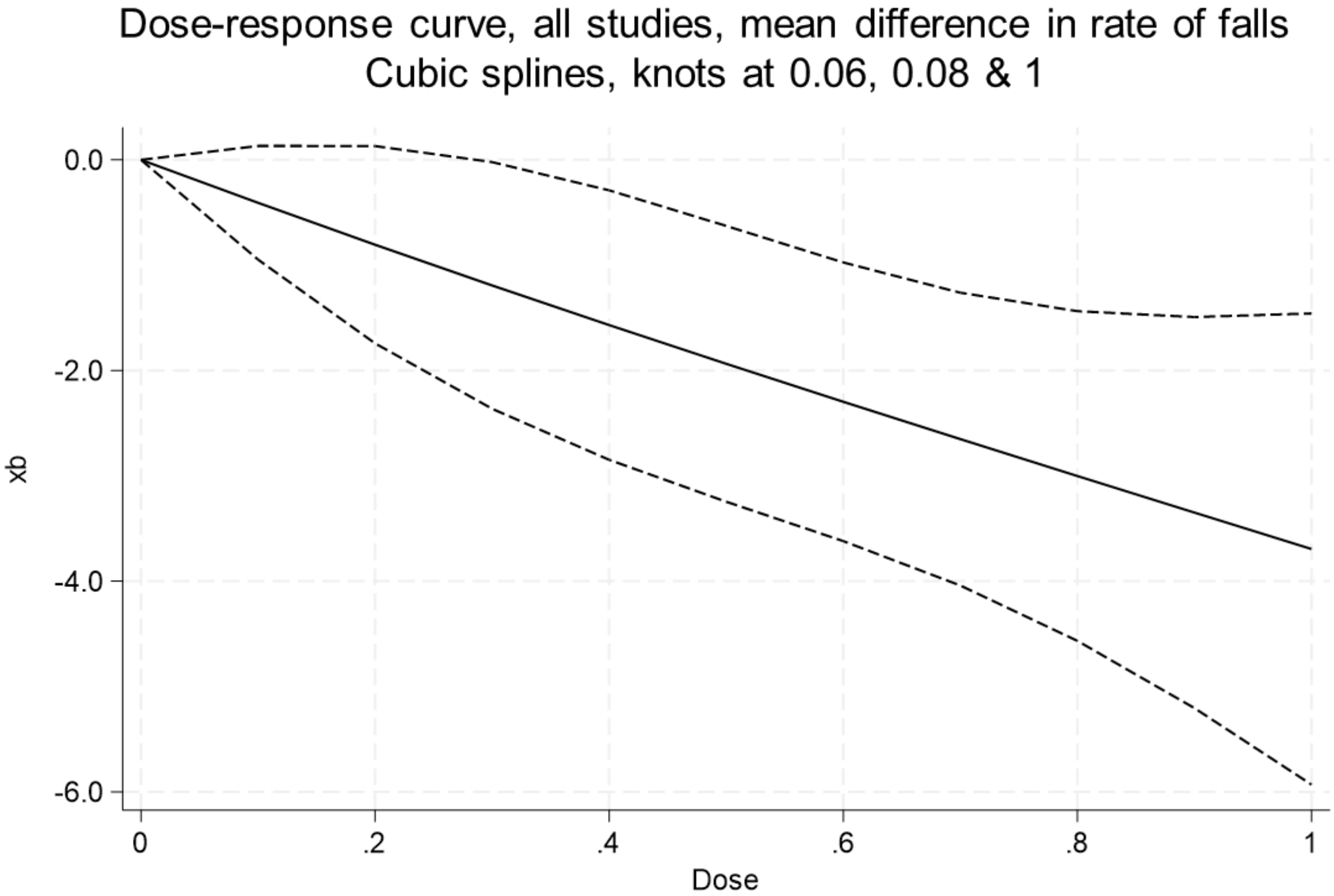

A significant dose-response model could not be identified when restricting analyses to just the RCTs for rate of falls (IRR or mean difference). A significant dose-response model could not be constructed when using all studies or just RCTs for rate of injurious falls, or odds ratio of becoming a faller.

Supplementary Material S10: Time-series data analyses.

i) Control / eliminated use of alarms – Prospectively registered standardised falls rate

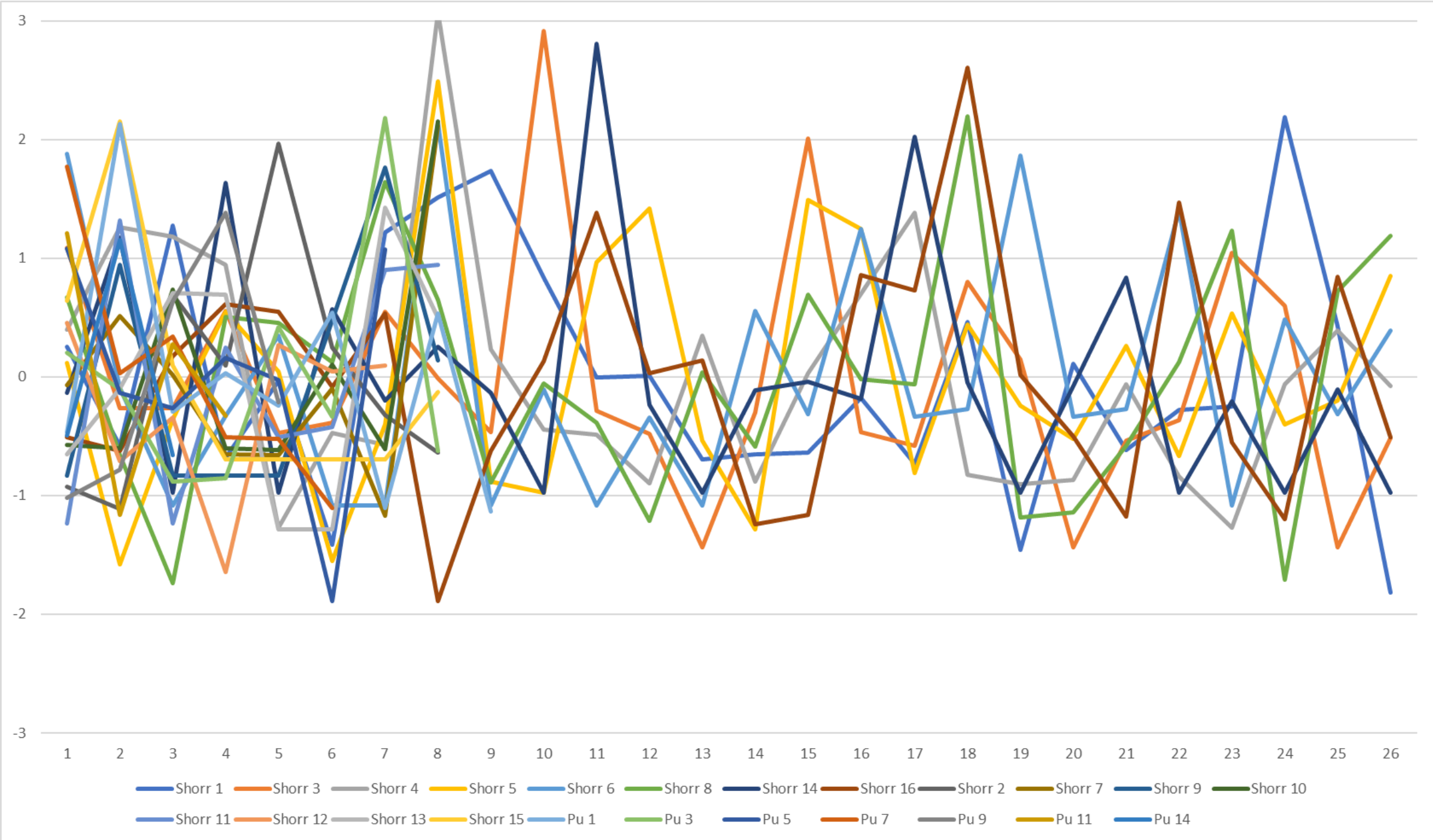

ii) Control / eliminated use of alarms – Prospectively registered standardised falls with injury rate

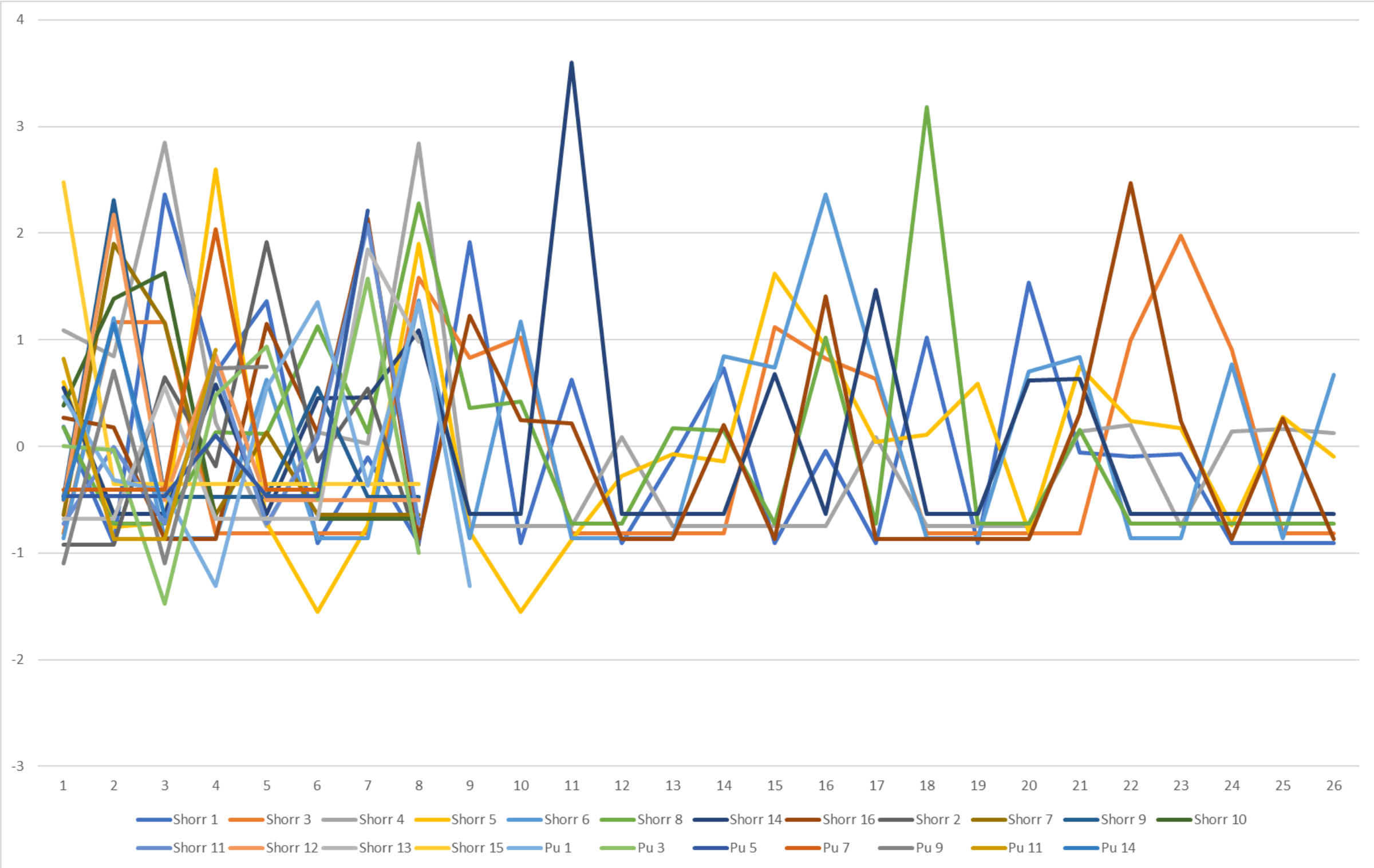

iii) Intervention / high use of alarms – Prospectively registered standardised falls rate

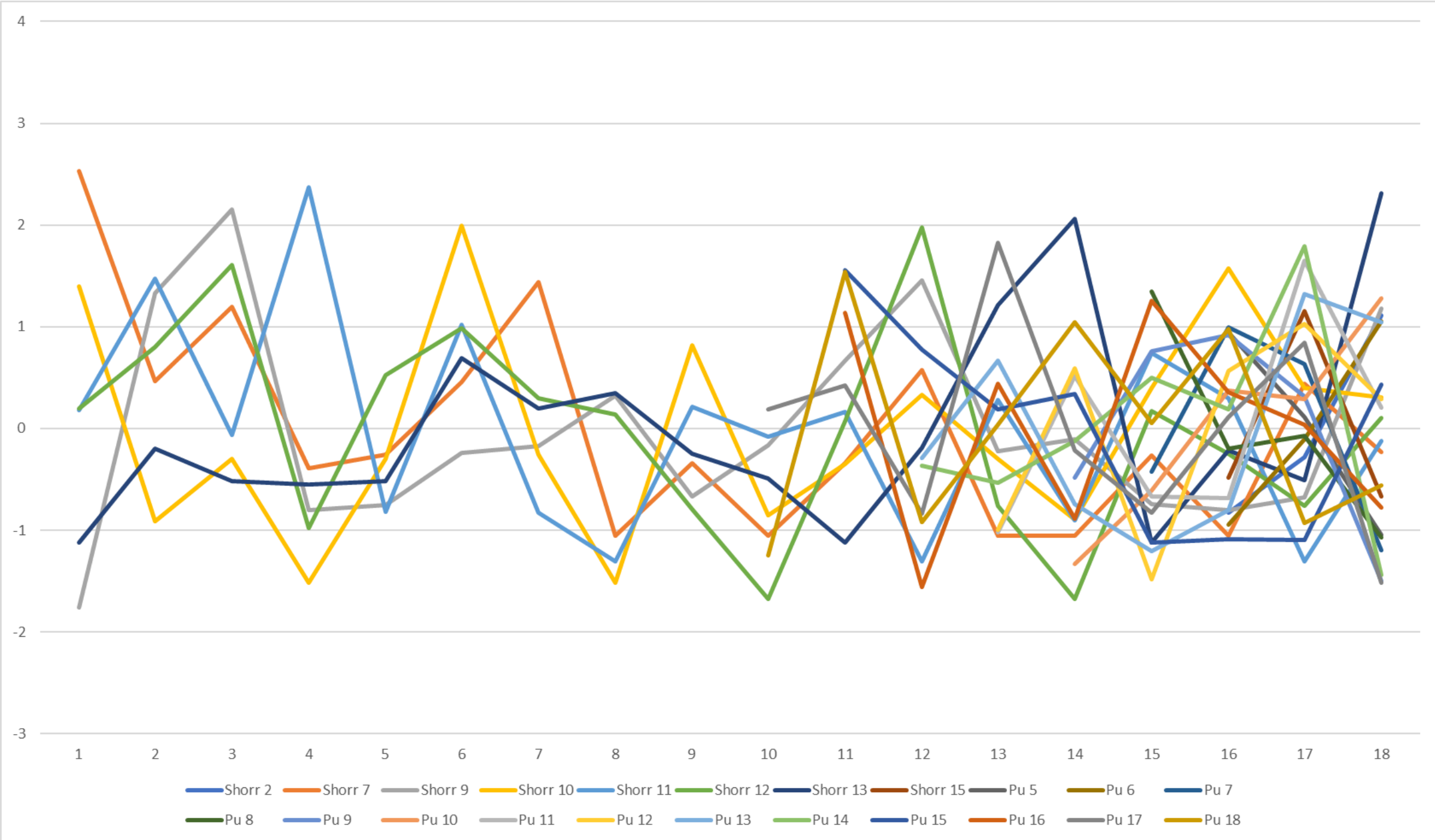

iv) Intervention / high use of alarms – Prospectively registered standardised falls with injury rate

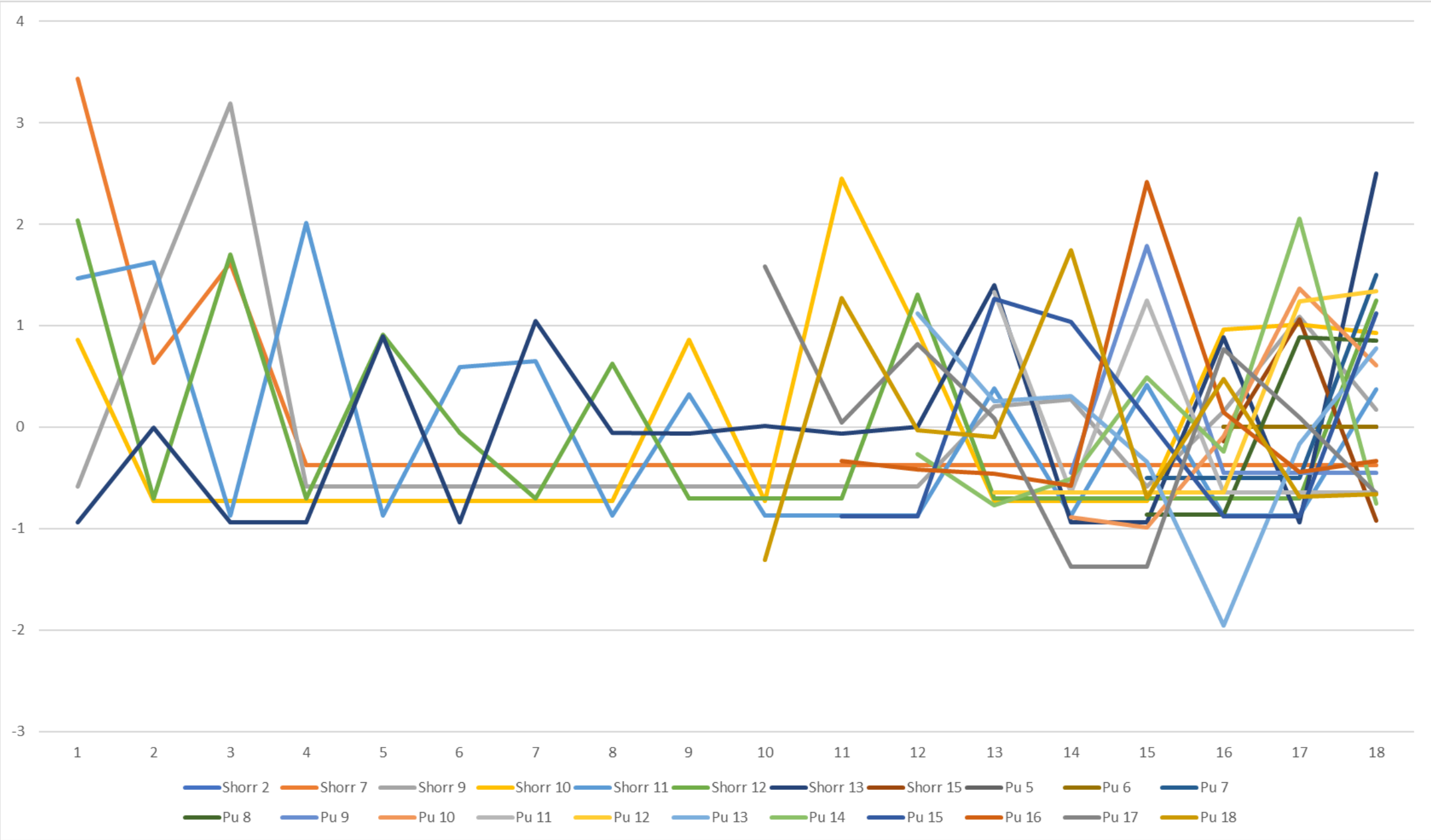

v) Control / eliminated use of alarms – Not prospectively registered standardised falls rate

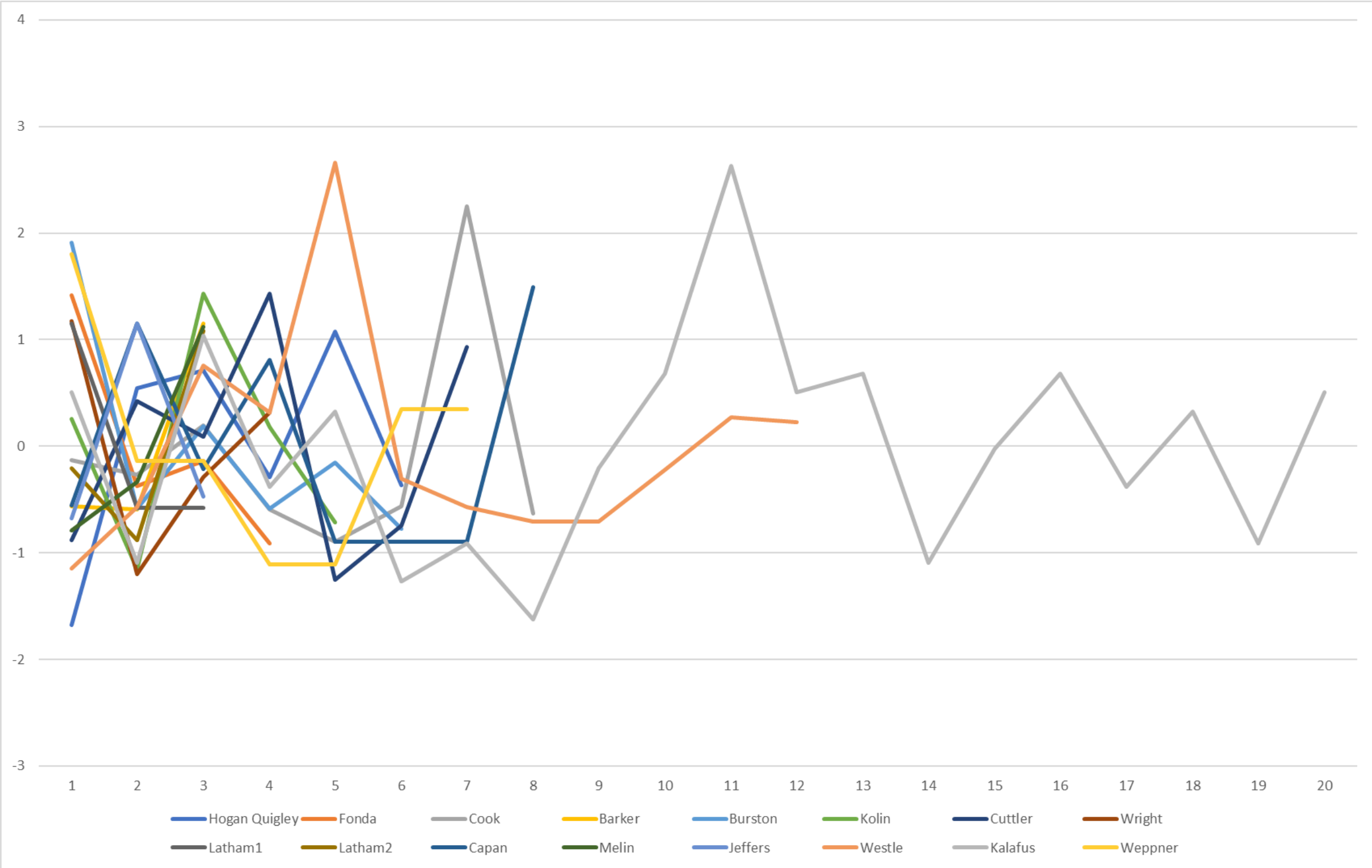

vi) Control / eliminated use of alarms – Not prospectively registered standardised falls with injury rate

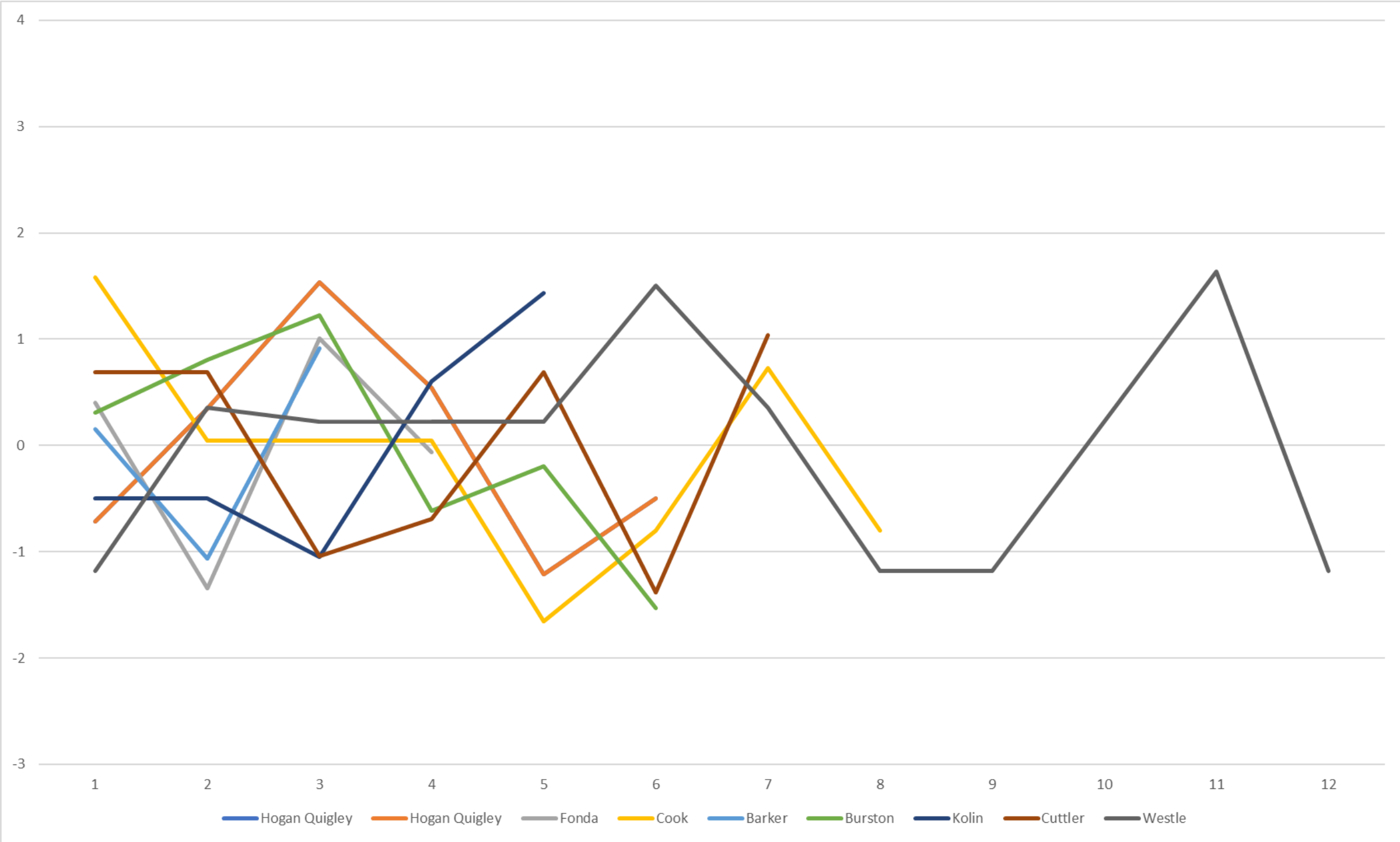

vii) Intervention / high use of alarms – Not prospectively registered standardised falls rate

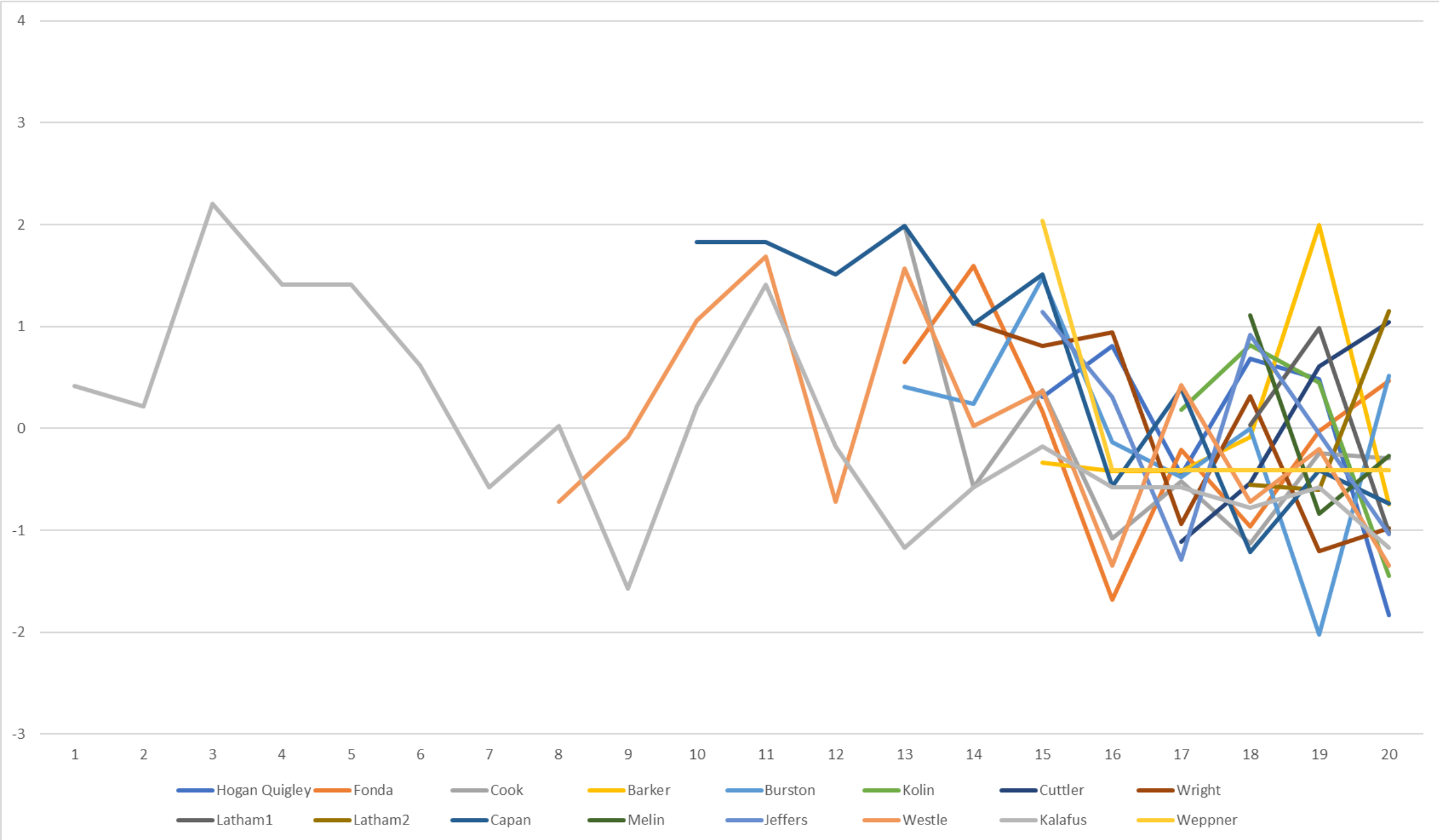

viii) Intervention / high use of alarms – Not prospectively registered standardised falls with injury rate

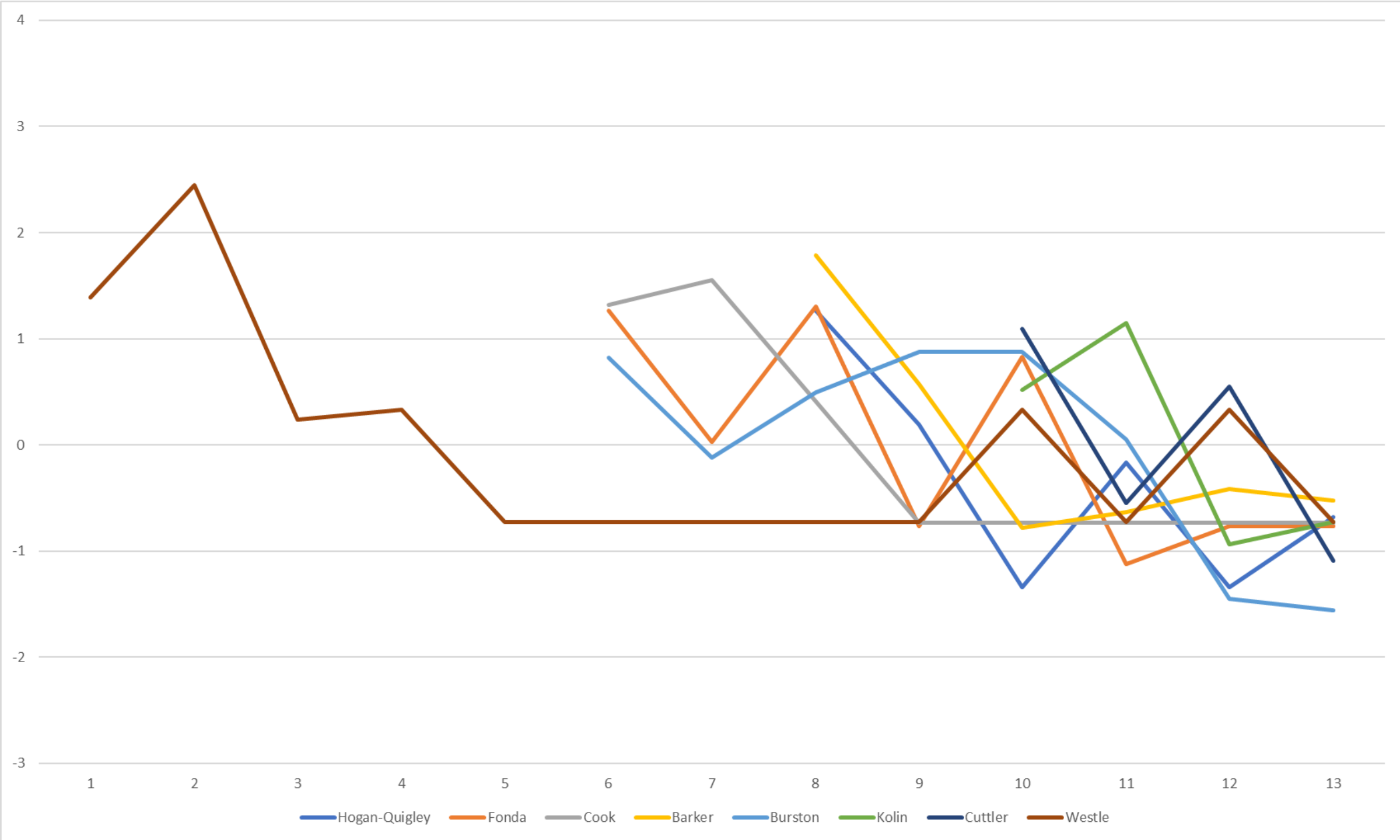

Supplementary Material S11. Comparison of standardised falls and falls with injury rate data from prospectively registered and non-prospectively registered studies at beginning of control / eliminated alarms condition or end of intervention / high alarms use conditions.

Outcome	Data point(s)	Prospectively registered	Not prospectively registered	Comparison of prospectively registered vs not prospectively registered
		Mean of standardised values (95%CI), [n units/wards, n studies]		Mean difference in standardised values (95%CI), [n hospitals/wards, n studies]
Rate of falls	First control data point	0.12 (-1.44 to 1.69), [23, 2]	0.10 (-0.49 to 0.69), [16, 15]	-0.02 (-0.64 to 0.59), [39, 17]
	Mean of first three control data points	0.03 (-0.72 to 0.78), [22, 2]	0.09 (-0.09 to 0.28), [11, 11]	0.06 (-0.14 to 0.26), [33, 13]
	Last intervention data point	-0.04 (-5.08 to 5.01), [22, 2]	-0.51 (-0.95 to -0.06), [16, 15]*	-0.47 (-1.23 to 0.29), [38, 17]
	Mean of last three intervention data points	0.06 (-0.15 to 0.28), [18, 2]	-0.32 (-0.57 to -0.08), [13, 13]*	-0.39 (-0.64 to -0.14), [31, 15]**
	First control data point	-0.07 (--1.44 to 1.30), [23, 2]	0.09 (-0.64 to 0.82), [8, 8]	0.16 (-0.56 to 0.89), [31, 10]

Rate of falls with injury	Mean of first three control data points	0.06 (-2.97 to 3.09), [22, 2]	0.14 (-0.31 to 0.60), [7, 7]	0.08 (-0.52 to 0.68), [29, 9]
	Last intervention data point	0.30 (-0.75 to 1.35), [22, 2]	-0.85 (-1.12 to -0.58), [8, 8]***	-1.15 (-1.45 to -0.85), [30, 10]***
	Mean of last three intervention data points	0.07 (-1.26 to 1.40), [18, 2]	-0.60 (-0.83 to -0.36), [8, 8]**	-0.67 (-0.96 to -0.38), [26, 10]**

* p<0.05

** p<0.01

*** p<0.001
